# Functional noninvasive detection of glycolytic pancreatic ductal adenocarcinoma

**DOI:** 10.1186/s40170-022-00298-5

**Published:** 2022-12-09

**Authors:** Irina Heid, Corinna Münch, Sinan Karakaya, Smiths S. Lueong, Alina M. Winkelkotte, Sven T. Liffers, Laura Godfrey, Phyllis F. Y. Cheung, Konstantinos Savvatakis, Geoffrey J. Topping, Florian Englert, Lukas Kritzner, Martin Grashei, Andrea Tannapfel, Richard Viebahn, Heiner Wolters, Waldemar Uhl, Deepak Vangala, Esther M. M. Smeets, Erik H. J. G. Aarntzen, Daniel Rauh, Wilko Weichert, Jörg D. Hoheisel, Stephan A. Hahn, Franz Schilling, Rickmer Braren, Marija Trajkovic-Arsic, Jens T. Siveke

**Affiliations:** 1 Institute of Diagnostic and Interventional Radiology, School of Medicine, Klinikum Rechts der Isar, Technical University of Munich, Munich, Germany; 2 West German Cancer Center, Bridge Institute of Experimental Tumor Therapy, University Hospital Essen, University of Duisburg-Essen, Essen, Germany; 3 Division of Solid Tumor Translational Oncology, German Cancer Consortium (DKTK, Partner Site Essen) and German Cancer Research Center, DKFZ, Heidelberg, Germany; 4 German Cancer Consortium (DKTK), Partner Site Essen, Essen, Germany; 5 Department of Nuclear Medicine, School of Medicine, Klinikum Rechts der Isar, Technical University of Munich, Munich, Germany; 6 Institute of Pathology, Ruhr University of Bochum, Bochum, Germany; 7 Department of Surgery, Knappschaftskrankenhaus, Ruhr University Bochum, Bochum, Germany; 8 Department of Visceral and General Surgery, St. Josef-Hospital, Dortmund, Germany; 9 Clinic for General and Visceral Surgery, St. Josef-Hospital, Ruhr-University Bochum, Bochum, Germany; 10 Department of Medicine, Ruhr University Bochum, University Hospital Knappschaftskrankenhaus Bochum GmbH, Bochum, Germany; 11 Medical Imaging, Radboud University Medical Center, Nijmegen, The Netherlands; 12 Faculty of Chemistry and Chemical Biology, TU Dortmund University, Dortmund, Germany; 13 Drug Discovery Hub Dortmund (DDHD) Am Zentrum Für Integrierte Wirkstoffforschung (ZIW), Dortmund, Germany; 14 Institute of Pathology, TUM School of Medicine, Technical University of Munich, Munich, Germany; 15 German Cancer Consortium (DKTK), Partner Site Munich, Munich, Germany; 16 Comprehensive Cancer Center Munich (CCCM), Munich, Germany; 17 Division of Functional Genome Analysis, German Cancer Research Center, DKFZ, Heidelberg, Germany; 18 Department of Molecular GI Oncology, Faculty of Medicine, Ruhr University Bochum, 44780 Bochum, Germany

**Keywords:** Glycolysis, PDAC, Lactate, Molecular subtype, Hyperpolarized magnetic resonance spectroscopy

## Abstract

**Background:**

Pancreatic ductal adenocarcinoma (PDAC) lacks effective treatment options beyond chemotherapy. Although molecular subtypes such as classical and QM (quasi-mesenchymal)/basal-like with transcriptome-based distinct signatures have been identified, deduced therapeutic strategies and targets remain elusive. Gene expression data show enrichment of glycolytic genes in the more aggressive and therapy-resistant QM subtype. However, whether the glycolytic transcripts are translated into functional glycolysis that could further be explored for metabolic targeting in QM subtype is still not known.

**Methods:**

We used different patient-derived PDAC model systems (conventional and primary patient-derived cells, patient-derived xenografts (PDX), and patient samples) and performed transcriptional and functional metabolic analysis. These included RNAseq and Illumina HT12 bead array, in vitro Seahorse metabolic flux assays and metabolic drug targeting, and in vivo hyperpolarized [1-^13^C]pyruvate and [1-^13^C]lactate magnetic resonance spectroscopy (HP-MRS) in PDAC xenografts.

**Results:**

We found that glycolytic metabolic dependencies are not unambiguously functionally exposed in all QM PDACs. Metabolic analysis demonstrated functional metabolic heterogeneity in patient-derived primary cells and less so in conventional cell lines independent of molecular subtype. Importantly, we observed that the glycolytic product lactate is actively imported into the PDAC cells and used in mitochondrial oxidation in both classical and QM PDAC cells, although more actively in the QM cell lines. By using HP-MRS, we were able to noninvasively identify highly glycolytic PDAC xenografts by detecting the last glycolytic enzymatic step and prominent intra-tumoral [1-^13^C]pyruvate and [1-^13^C]lactate interconversion in vivo.

**Conclusion:**

Our study adds functional metabolic phenotyping to transcriptome-based analysis and proposes a functional approach to identify highly glycolytic PDACs as candidates for antimetabolic therapeutic avenues.

**Supplementary Information:**

The online version contains supplementary material available at 10.1186/s40170-022-00298-5.

## Background

Despite enormous research efforts, pancreatic ductal adenocarcinoma (PDAC) remains a fatal disease with marginal clinical advancement [1]. Although genomic and transcriptional profiles of PDAC have been studied in great detail [2–4], effective targeting strategies remain scarce. Sequencing efforts in large patient cohorts have identified distinct molecular PDAC subtypes in several independent studies with two dominant subgroups. Those are termed classical (term used hereafter) or pancreatic progenitor with more epithelial differentiated tumor and quasi-mesenchymal (QM; term used hereafter) or squamous or basal like [1, 5–7] with more mesenchymal tumor respectively. Especially, QM PDACs demonstrate very aggressive phenotypes with shorter median survival and resistance to first-line chemotherapy with FOLFIRINOX [1]. Which cancer cell features contribute to the aggressive and therapy-resistant QM phenotype remains unknown.

Metabolic rewiring, i.e., an individual cell’s ability to use different metabolic pathways depending on alternating growth conditions including oxygen and nutrient availability, has been implicated as a major cause of therapy resistance in cancers and aggravates clinically successful targeting [8]. This allows cells not only to adapt but also to thrive on particularly scarce conditions of hypoxia and nutrient limitations typically observed in PDAC [9]. Glycolysis is the most prominent cancer-associated metabolic pathway. Although high cancer dependency on glucose was described nearly 100 years ago by Otto Warburg [10], glycolytic targeting is still not widely therapeutically exploited. Recently, expression of glycolytic metabolic transcripts has been associated with the resistant QM PDAC subtype in patients [11]. Work in PDAC mouse models demonstrated that glycolysis is the major metabolic effector of oncogenic KRAS, the leading PDAC driver, and that co-targeting of RAS-RAF-MEK-MAPK cascade and glycolysis may be an effective approach in PDAC [12]. However, functional evidence that glycolysis is indeed significantly operable in human QM PDACs is missing.

Here, we addressed this missing link and analyzed functional exposure of glycolysis in different clinically relevant PDAC samples ranging from long-term cultured PDAC cell lines to patient-derived xenografts and primary cells and bulk PDAC probes. We found considerable heterogeneity in the glycolytic behavior especially among patient-derived PDAC cells. However, individual representatives of the QM subtype were indeed functionally highly glycolytic what was preserved even in the in vivo xenograft setting. By using a noninvasive hyperpolarized ^13^C-magnetic resonance spectroscopy (HP-MRS), we were able to detect the final glycolytic step in vivo, namely intratumoral conversion of HP-[1-^13^C]pyruvate to HP-[1-^13^C]lactate. Importantly, QM PDAC cells actively consumed the final glycolytic product, lactate, in mitochondrial oxidative phosphorylation in vitro, what was further in vivo translated and detected as HP-[1-^13^C]lactate to HP-[1-^13^C]pyruvate conversion in QM PDAC xenografts. This suggested that glycolytic QM cells not only actively produce lactate but also metabolically use it. Our work opens a perspective for noninvasive detection of glycolytic PDACs and monitoring of individualized anti-glycolytic targeting approaches.

## Methods

### PDAC cell lines

All PDAC cell lines have been obtained from the ATCC and regularly externally authenticated (at least once a year). PDAC cell lines (PSN1 (RRID: CVCL_1644); Kp4 (RRID: CVCL_1338), PaTu8988T (CVCL_1847); MiaPaca2 (RRID: CVCL_0428), PaTu8988S (RRID: CVCL_1846), HPAC (RRID: CVCL_3517), HPAFII (RRID: CVCL_0313), and HupT4 (RRID: CVCL_1300) were grown in Dulbecco’s Modified Eagle Medium (1:1 mix of DMEM no. 11966025 and DMEM no. A1443001, Thermo Fisher Scientific, Waltham, USA) adapted to final concentrations of 5 mM D-glucose (Thermo Fisher Scientific, Waltham, USA), 2 mM L glutamine, 5% v/v fetal bovine serum (FBS, Thermo Fisher Scientific, Waltham, USA), and 1% v/v penicillin/streptomycin (P/S, Thermo Fisher Scientific, Waltham, USA) if not stated otherwise.

### Patient-derived cells (PDCs)

From 11 PDX samples, we were able to isolate and cultivate cancer cells (PDCs) for further analysis. For all metabolic analysis, PDC cell lines were cultivated in a 1:1 mixture of Keratinocyte-SF medium (no. 17005075, Thermo Fisher Scientific, Waltham, USA) and RPMI 1640 (no. 11879020, Thermo Fisher Scientific, Waltham, USA) adapted to final concentrations of 5 mM D-glucose, 4.5 mM L-glutamine, 0.26 mM sodium pyruvate, 6%v/v FBS, and 1% v/v final mixture of penicillin/streptomycin (Thermo Fisher Scientific, Waltham, USA), and antimycotic/antibiotic (cat. no. 15240–062, Thermo Fisher Scientific) if not stated otherwise.

### RNA isolation and gene expression analysis

Established/PDC cells were cultivated for 48 h in the respective media. At confluence of 70–80%, cells were placed on ice and washed twice with ice-cold PBS, mechanically scratched from the plate in 1 ml of ice-cold PBS, and centrifuged at 4 °C/400 g for 5 min. Pelleted cells were stored in − 80 °C until all cells were collected for RNA isolation. RNA was isolated using the Maxwell RSC simplyRNA Cells Kit (no. AS1390, Promega, Germany). Cell RNA isolation kit was used according to the manufacturer’s instructions. Total RNA was stored at − 80 °C until further processing and gene expression analysis. For PDX samples, RNA was isolated from fresh-frozen PDX tumor tissue using the PARIS (Ambion) isolation kit.

### PDX samples preparation

Establishment of the PDX mouse model was performed using surgically resected PDAC tissues collected from patients.

### Seahorse metabolic flux assays

All assays were performed following the manufacturer’s instructions (Agilent Technologies).

### Immunohistochemistry and immunofluorescence

Immunohistochemistry was performed according to standard laboratory procedures on PFA fixed, FFPE tissue samples. Antibodies used in this study are as follows: MCT4, Atlas Antibodies (Sigma-Aldrich, Cat no. HPA021451, RRID:AB_1853663); HIF1a, BD Transduction laboratories no. 610959 (RRID: AB_398272); MCT1, Abcam, no. ab85021(RRID: AB_10674945); KRT81, Santa Cruz, no. sc-100929 (RRID: AB_2132772); and pancytokeratin, Abcam no. ab6401(RRID: AB_305450).

### Hyperpolarized magnetic resonance spectroscopy (HP-MRS)

PSN1/HPAC cells were implanted subcutaneously (s.c.) into the back of male or female 6-week-old Crl:NIH-Foxn1^rnu^ rats (Charles River). Pyruvate-lactate metabolism was measured with multi-frame slice spectroscopy (MRS, 15 mm slice thickness) using alternating metabolite-frequency-selective excitation (flip angle 30°, 250 Hz transmit bandwidth, 2 kHz receiver bandwidth, both metabolites separately excited and measured every 2 s) while injecting hyperpolarized HP-[1-^13^C]pyruvate or HP-[1-^13^C]lactate. Procedure optimization is described in detail in our previous study [13]. For data analysis after HP-[1-^13^C]pyruvate injection, lactate and pyruvate spectral peak heights were summed over all time points and presented as the ratio of these areas under curves (AUC_lac_/AUC_pyr_) [14]. For data analysis after HP-[1-^13^C]lactate injection, due to low pyruvate signal, the signal intensities of lactate and pyruvate spectra were averaged over 10 time points near the maximum intensity and then fit with a constant offset plus a Lorentzian function with fixed 30 Hz full width at half maximum to determine the peak area (PA) [15], using the least-squares curve fit function in MatLab.

## Results

### Glycolytic metabolic gene transcripts are present in QM PDAC subtype

**Fig. 1:**
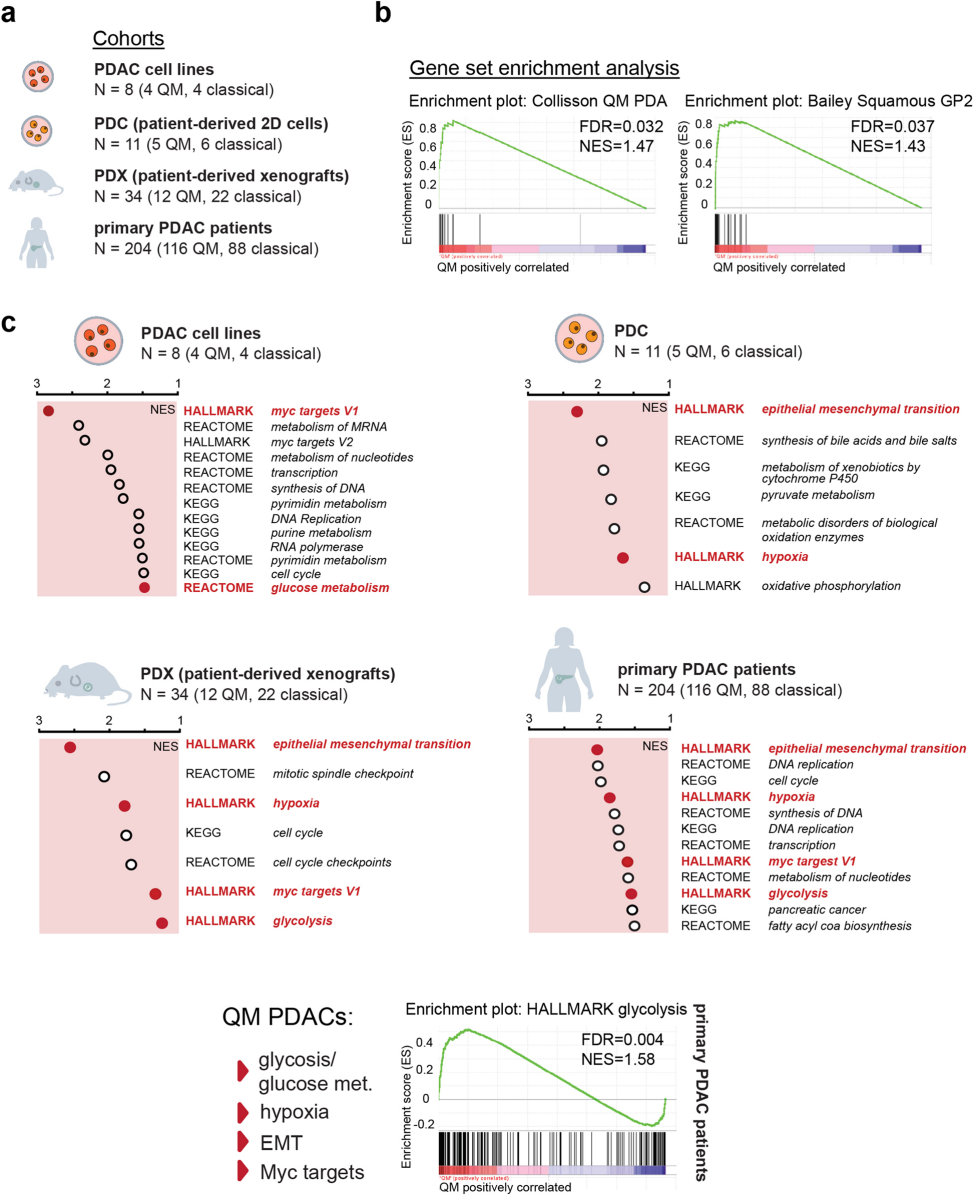
Gene set enrichment analysis (GSEA) in different PDAC cohorts and models. **a** Models used in this study. **b** Enrichment plots for the selected “Collisson QM” and “Bailey squamous GP2” assigner gene sets in our patient cohort. Both gene sets are enriched in here defined QM PDAC samples. FDR (false discovery rate) and NES (normalized enrichment score) presented in the figure. **c** GSEA analysis for QM vs classical groups was performed for cell lines (*n* = 8; 4QM, 4 classical), patient-derived cells (PDC; *n* = 11, 5 QM and 6 classical), patient-derived xenografts (PDX; *n* = 34, 12 QM and 22 classical), and patient PDAC samples (*n* = 204; 116 QM, 88 classical). Presented are NES values for selection of metabolic gene sets identified as enriched (*NES* > 1.3, FDR *q*-value < 0.07) in QM subtype. The gene set databases HALLMARK, REACTOME, and KEGG were used for analysis. Epithelial-to-mesenchymal transition (EMT), glycolysis/glucose metabolism, hypoxia, and MYC targets gene sets are commonly enriched in QM datasets. Red dots emphasize the metabolic pathways that are commonly enriched in the models presented here. Glycolysis enrichment plot for patient cohort (*n* = 204) presented

To evaluate whether glycolytic transcripts are omnipresent in the QM PDAC subtype [11], we first performed gene expression analysis in multiple preclinical and clinical samples (Fig. 1a). RNA-seq or Illumina HT12 gene expression analysis was performed for conventional PDAC cell lines (*n* = 8), patient-derived xenografts (PDX, *n* = 34), and PDX-derived cancer cells (PDC, *n* = 11). Transcriptomes from bulk tissue of 204 PDAC samples from previously published resource were utilized (E-MTAB-1791). All samples were then subtyped to QM or classical group. For tumor subtype determination, nonnegative matrix factorization (NMF) [16] was used, after median centering of the data. ConsensusClusterPlus [17] was used to validate class assignment from NMF. In a first benchmarking step, we used publicly available transcriptionally subtyped PDAC cohorts (PDAC cell lines (GSE21654 [18]), PDAC xenograft (E-MTAB-4029 [19]), and bulk PDAC tissue (GSE16515 and GSE15471 [20, 21]) to verify the robustness of our classification pipeline. With this approach, we were able to reproduce more than 90% of the reported subtypes in our test datasets.

After establishing the pipeline, the same parameters were used for subtyping of patient PDAC samples, PDX cohort, and PDC samples to QM, and classical group and gene set enrichment analysis (GSEA) comparing the QM and classical groups were performed. By using our subtyping platform, in the PDAC patient sample cohort (204 samples), 88 are classified as classical and 116 as QM subtype. Samples clustering to the QM subtype presented significant enrichment of selected QM and squamous subtype assigner gene sets previously described [5, 6] (Fig. 1b and supplementary Table 1) supporting correct subtype assignment.

In the PDX cohort, 22 classical and 12 QM tumors were identified. Among PDCs, 6 classical (PDC44, 58, 59, 62, 70, 89) and 5 QM (PDC 34, 57, 69, 78, 80) were identified. The 8 PDAC cell lines used in this study were previously classified as QM (KP4, PSN1, MIAPaca2, PaTu8988T) and classical (PaTu8988S, HUPT4, HPAFII, HPAC) [22]. We analyzed gene expression of vimentin (*VIM*) and E-cadherin (*CDH1*) as markers of mesenchymal and epithelial status respectively. As expected, general trend towards higher *VIM* expression in QM and *CDH1* expression in classical PDAC cells was observed (supplementary Figure. 1a).

After classification, QM and classical groups were compared by GSEA for HALLMARK, REACTOME, and KEGG collections in all datasets. A full list of all enriched gene sets with respective normalized enrichment score (NES) and false discovery rate (FDR) values is given in supplementary Table 2. As expected for the mesenchymal phenotype, enrichment of the epithelial-to-mesenchymal transition (EMT) gene set was observed in the QM group in PDX, PDC, and patient PDAC samples (Fig. 1c) supporting correct assignment of the subtypes. In the QM samples, transcripts for glycolysis, hypoxia, and MYC-target genes were well preserved throughout different sample collections (Fig. 1c). The hypoxia gene set was enriched in QM bulk PDAC tissue, PDX, and PDC data sets, even though PDC cells were cultured under common laboratory normoxic conditions. Concordantly with the well-described correlation of hypoxia and glycolysis [23], glycolysis/glucose metabolism transcripts were also enriched in the QM patient PDAC samples, PDX and PDAC cell line datasets, and MYC target gene sets as well. Interestingly, in the QM PDCs, the glycolysis gene set was not enriched, possibly due to low sample numbers but also suggesting no unambiguous assignment of glycolytic genes to the QM subtype at least in PDCs. In summary, we observed strong transcriptional association of QM subtype with glycolysis in different preclinical and clinical samples.

### Glycolytic pathway activity is high in individual QM PDAC cells

**Fig. 2:**
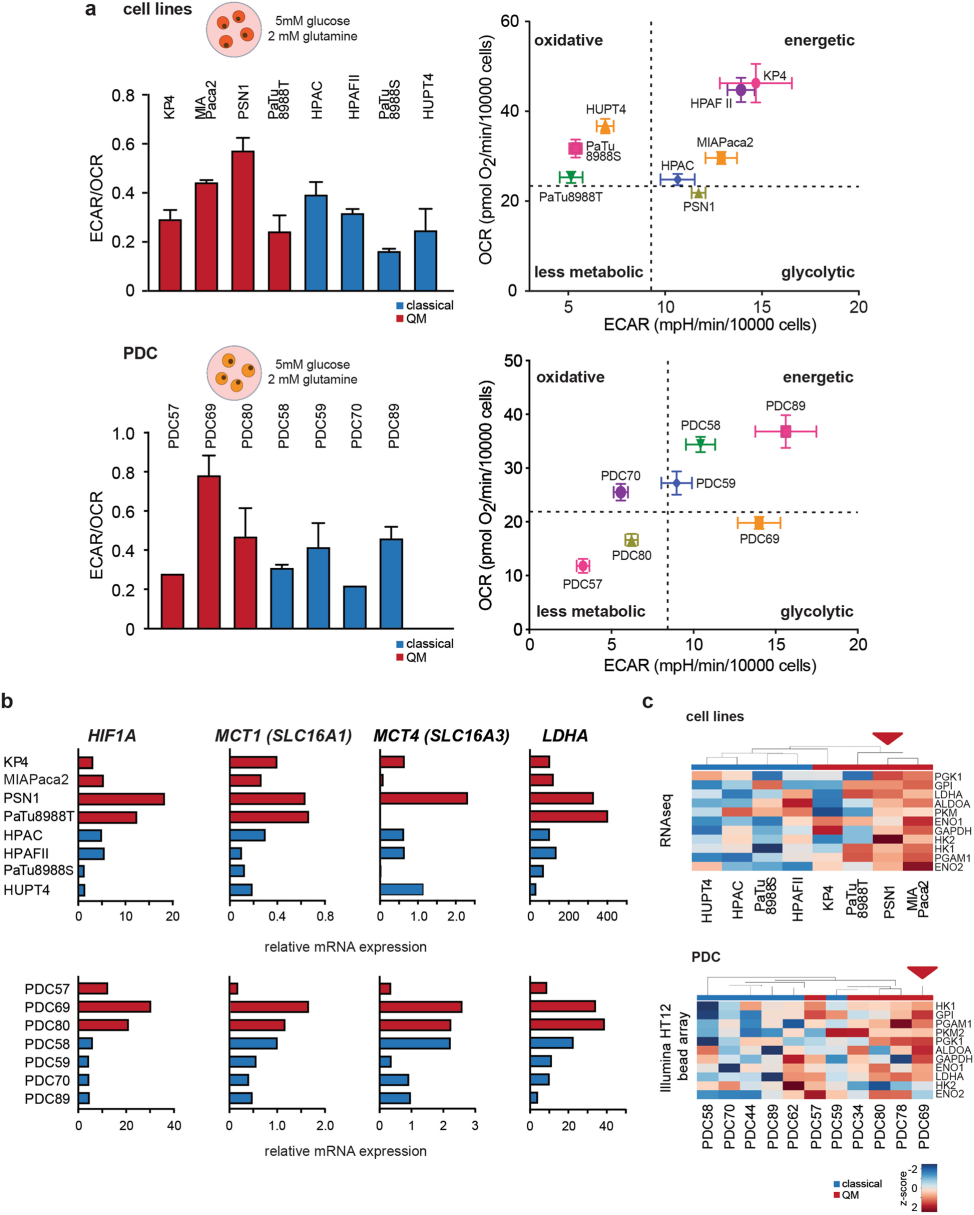
Functional glycolysis evaluation in PDAC cells. **a** ECAR to OCR ratios (ECAR/OCR) and energy maps as measured by seahorse metabolic flux assay for PDAC cell lines (upper) and PDCs (lower) in medium supplemented with 5 mM glucose (physiological concentration) and 2 mM glutamine. Higher ECAR/OCR ratio indicates higher glycolysis in PSN1 and PDC69 cells under these conditions. Presented are mean + SD values calculated from 2 independent experiments, with at least 5 technical replicates per cell line per experiment. Energy maps (OCR vs ECAR plots) show glycolytic energetic positioning of PSN1 and PDC69 cells. Representative energy maps from one experiment, at least 5 technical replicates per cell line. At least 2 independent experiments performed. OCR and ECAR values were normalized to 10,000 post-experimentally counted, viable cells. Dotted lines present arbitrary cutoff levels used for separation of different energy phenotypes (glycolytic, oxidative, energetic, or less metabolic). **b** Relative gene expression (qPCR) data for *LDHA*, MCT1 (*SLC16A1*), MCT4 (*SLC16A3*), and *HIF1a* in cell lines and PDCs. High gene expression levels were observed for PSN1 and PDC69 (both QM subtype). Beta-glucuronidase (GUSB) expression was used as house-keeper control. **c** Hierarchical clustering analysis for glycolytic genes using gene expression data for cell lines (RNA-seq) and PDCs (HT12 Illumina gene expression array). Z-score: red color—high expression, blue color—low expression. PSN1 and PDC69 show higher expression of investigated glycolytic genes

To investigate whether glycolysis is indeed functionally active in QM PDAC cells, we performed Seahorse metabolic flux assays and evaluated the extracellular acidification rate (ECAR) and oxygen consumption rate (OCR) as readouts of two major energy-supplying processes, glycolysis and oxidative phosphorylation respectively. ECAR and OCR levels were measured in cell lines and PDCs in media with physiological concentrations of 5 mM glucose with addition of 2 mM glutamine. Under these conditions, PSN1 and PDC69, both QM, presented the highest ECAR/OCR ratios and glycolytic energy phenotype among cell lines and PDCs, respectively (Fig. 2a and supplementary Table 3). However, energetic phenotype of other cell lines and PDCs was rather heterogeneous and independent of their transcriptional subtype, being QM or classical.

Notably, qPCR analysis revealed that the relative gene expression of the last glycolytic enzyme lactate dehydrogenase A (*LDHA*), lactate exporter MCT4 (*SLC16A3*), and importer MCT1 (*SLC16A1*) is high in PSN1 and PDC69 cells in comparison with other cells. The same was true for *HIF1a*, a central cellular regulator of hypoxia and glycolysis (Fig. 2b). Moreover, hierarchical clustering of transcriptome data showed generally higher expression of several glycolytic genes (e.g., *HK1*, *HK2*, *ENO1*, *ENO2*, *PGK*1) in QM cell lines and PDCs, especially in PSN1 and PDC69 (Fig. 2c). Taken together, active glycolysis was observed in some QM PDAC cells that correlated well with the high expression of glycolytic genes but was not unambiguously connected to QM subtype.

**Fig. 3:**
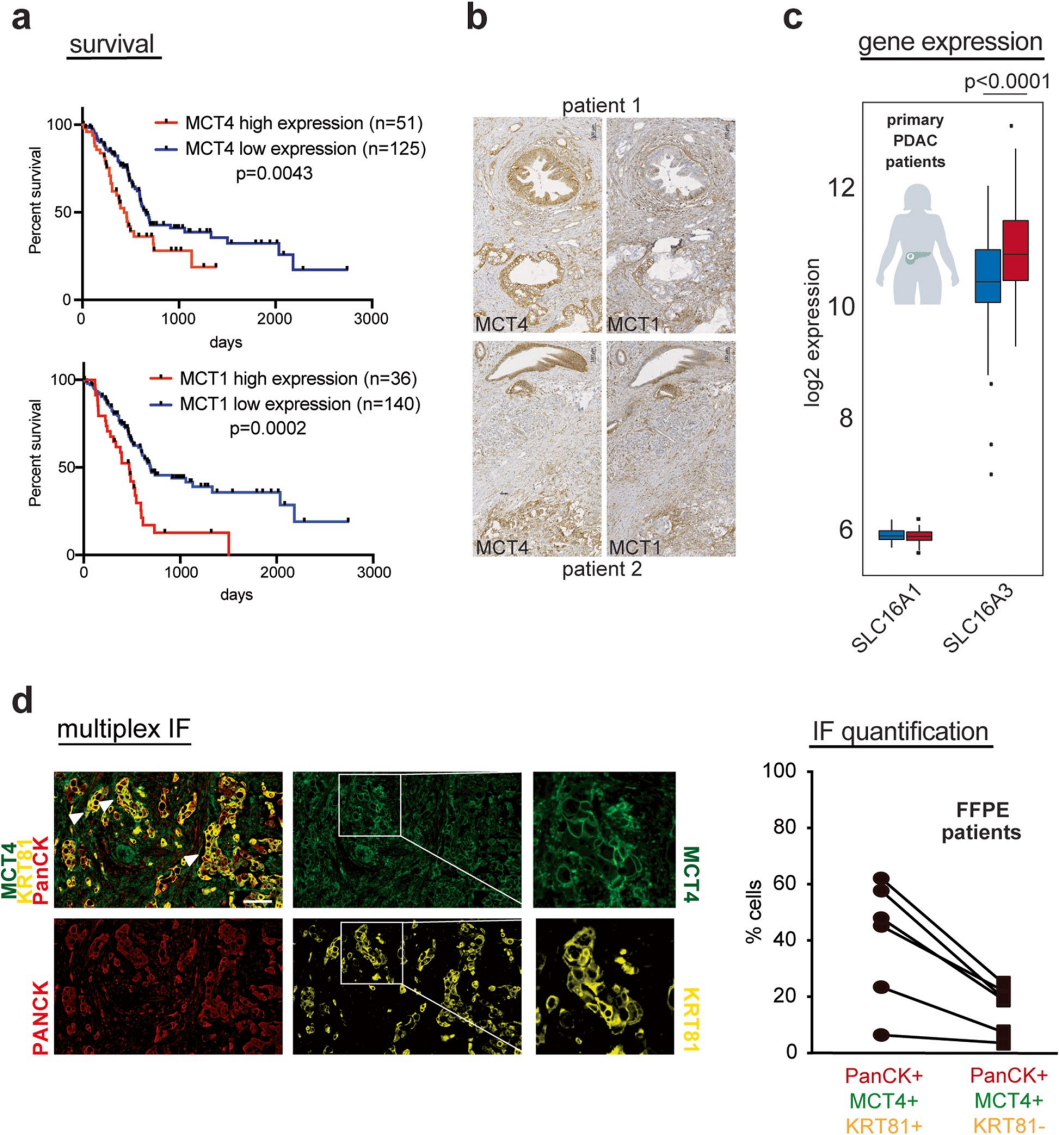
Functional glycolysis evaluation in PDAC cells. **a** Survival analysis of PDAC patients according to *SLC16A3* (MCT4) and SLC16A1 (MCT1) gene expression. Data from www.proteinatlas.org. Patients with higher MCT4 and MCT1 expression present worse survival. **b** Immunohistochemistry for MCT1 and MCT4 on patient FFPE PDAC samples emphasizing MCT4 and MCT1 expression on both cancer and stromal cells with prominent MCT4 expression in cancer and MCT1 expression in stroma cells. Scale bar, 100 µm. **c** *SLC16A1* (MCT1) and *SLC16A3* (MCT4) gene expression in patient bulk PDAC samples (*n* = 204; 116 QM, 88 classical) emphasizing higher expression of SLC16A3 than of SLC16A1. SLC16A3 is also significantly highly expressed in QM than in classical human PDAC bulk samples. *P*-value calculated by Student’s *T*-test (unpaired, two sided). **d** Multiplexed immunofluorescence staining of MCT4 (green), cytokeratin 81 (KRT81, QM marker—yellow), and pan-cytokeratin (PANCK, cancer cell marker—red) on *n* = 6 patient PDAC FFPE samples. White arrows indicate overlapping MCT4 and KRT81 signals. Scale bar: 10 µm. Right graph: quantification of respective populations in 6 PDAC samples by halo. A total of 30–50% of KRT81 + cancer cells are also MCT4 positive; among KRT81 − cancer cells, less than 20% are also positive for MCT4. Populations are determined in the same sample; one line indicates one patient

Lactate exporter MCT4 has previously been suggested to be a good marker of glycolytic PDACs [24]. In publicly available TCGA RNA expression datasets, high expression of both lactate transporters MCT4 and MCT1 correlated with worse survival in PDAC patients associated with the resistant QM subtype (Fig. 3a). Immunohistochemical analysis of MCT1 and MCT4 in FFPE samples of 30 human PDACs suggested that both MCT4 and MCT1 were expressed on cancer and stromal cells with MCT4 being more prominently expressed on cancer cells, while MCT1 was often prominently expressed in the surrounding stroma as well (Fig. 3b). In our cohorts, we observed that MCT4 (*SLC16A3*) gene expression levels were higher than MCT1 (*SLC16A1*) in both bulk PDAC and PDX tissue samples (Fig. 3c and supplementary Figure. 2a), suggesting a lead role of MCT4 as lactate transporter in tissue context. Furthermore, multiplex immunofluorescence for pancytokeratin (PanCK), MCT4 and KRT81, an established QM marker [19], in 6 PDAC FFPE specimens, showed that the proportion of MCT4-positive cells was higher among KRT81 positive (30–50%) than KRT81-negative cancer cells (< 20%) (Fig. 3d). Our data support the use of MCT4 as a surrogate marker of QM PDACs with activated glycolysis.

### PDAC cells actively use lactate as oxidative fuel

**Fig. 4:**
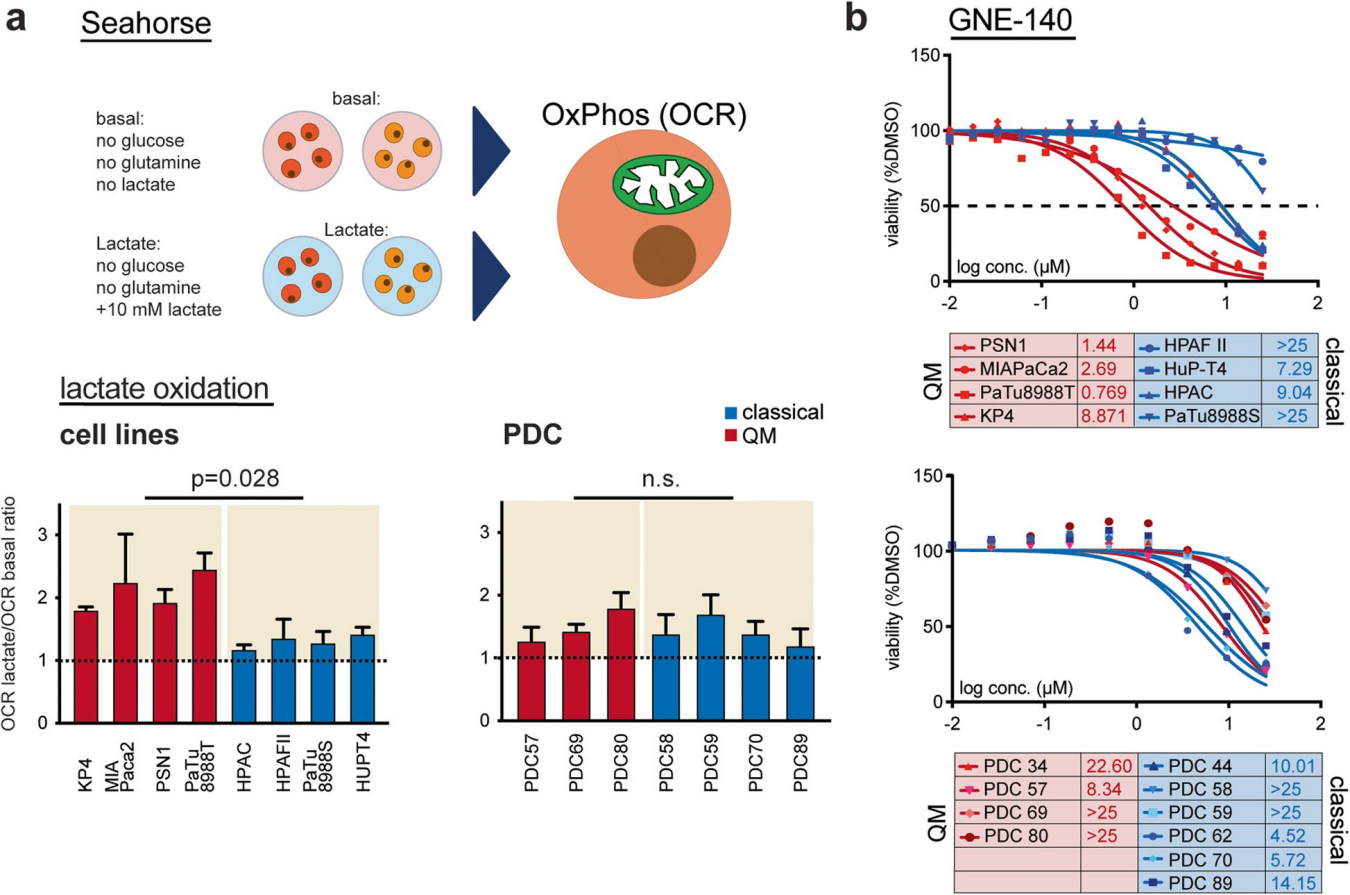
Lactate is used as oxidative fuel in PDAC cells. **a** Schematic representation of the performed Seahorse assay. Cells were cultivated in “basal” medium (no glucose, no glutamine) or in “basal” medium supplemented with 10 mM sodium-L-lactate (“basal + lactate”) for 7 h in total, and OCR levels are measured. Ratios among OCR values measured for “basal + lactate” and “basal” only media are calculated and presented. Ratio above 1 (dotted line) indicates increase in OCR after lactate application. Presented are mean ratios + SD values of minimum 2 independent experiments, at least 3 technical replicates per cell line/per condition/per experiment. *P*-values calculated by the Mann–Whitney test. **b** Dose–response curves of cell lines and PDCs to LDH inhibitor GNE-140. Presented are mean dose–response curves and IC50 values of two independent experiments; 3 technical replicates per concentration/cell line/experiment were performed. Stronger response observed in QM than in classical cell lines. No differences in response rate among QM and classical PDCs

Intrigued by high expression of lactate transporters detected in some of the QM PDAC cells, we aimed to investigate lactate metabolism in PDAC. It is now well accepted that lactate is not only the end waste product of glycolysis but is also actively used in metabolic processes in cancer as well. Lactate conversion to pyruvate and subsequent oxidation in the mitochondria has been suggested in murine PDAC [25]. However, whether this effect is especially attributable to lactate-producing high-glycolytic QM PDAC cells is still not known. To investigate this, PDAC cells were cultivated for 7 h in (i) “basal” DMEM or RPMI media without glucose or glutamine supplementation or in (ii) “basal” media supplemented with lactate (basal + 10 mM L-lactate). Consequently, Seahorse metabolic flux measurement was performed, and OCR values measured in media with and without lactate were compared. In basal media, classical PDAC cell lines (HPAC, HPAFII, and HupT4) presented generally higher basal OCR levels than the QM cell lines, indicating that oxidative phosphorylation (OXPHOS) is well supplied by alternative fuels other than glucose or glutamine in the classical cell lines (supplementary Figure 2b). Interestingly, lactate supplementation to the medium led to OCR boost in all cells of both QM, and classical subtypes with however more pronounced OCR increase in the QM PDAC cell lines (Fig. 4a). In PDCs, lactate treatment led to an OCR increase in all cells, without pronounced subtype-specific effect (Fig. 4a and supplementary Figure 2b).

To substantiate this finding, we cultivated PSN1 (QM), PaTu8988T (QM), and PaTu8988S (classical) cells in DMEM medium with 5 mM glucose and 2 mM glutamine without media change for 24–48 to 72–96 h. Glucose and lactate concentrations in the media were measured at given time points. With time, glucose concentration in the media decreased, and lactate increased (0–72 h), as expected due to glucose consumption and lactate production and accumulation. Once the glucose was consumed from the medium (approx. after 72 h in PaTu8988T/PSN1 cells), lactate concentration in the media decreased, indicating that in the absence of other resources, PDAC cells start consuming self-produced lactate (supplementary Figure 2c).

We also challenged the detected changes in glycolysis and lactate metabolism with the inhibitor of lactate dehydrogenase GNE-140 and followed the concentration-dependent inhibition of metabolic activity in cells via CellTiter-Glo assay (Fig. 4b). GNE-140 treatment indeed induced a decrease in cell viability especially in the QM cell lines, being most effective in PSN1, MIAPaca2, and PaTu8988T cells. PDCs were in general less sensitive to GNE-140, and the observed inhibitory effects were, as expected from Seahorse lactate supplementations assays, not subtype dependent.

In conclusion, PDAC cells, regardless of subtype, not only actively produce and excrete glycolytically produced lactate but also actively use it potentially as an oxidative fuel. This phenomenon is more pronounced in QM than in classical PDAC cell lines and is exposed to specific metabolic targeting with lactate dehydrogenase inhibitors.

### Hyperpolarized magnetic resonance spectroscopy of [1-^13^C]pyruvate and [1-^13^C] lactate identifies highly glycolytic tumors

**Fig. 5:**
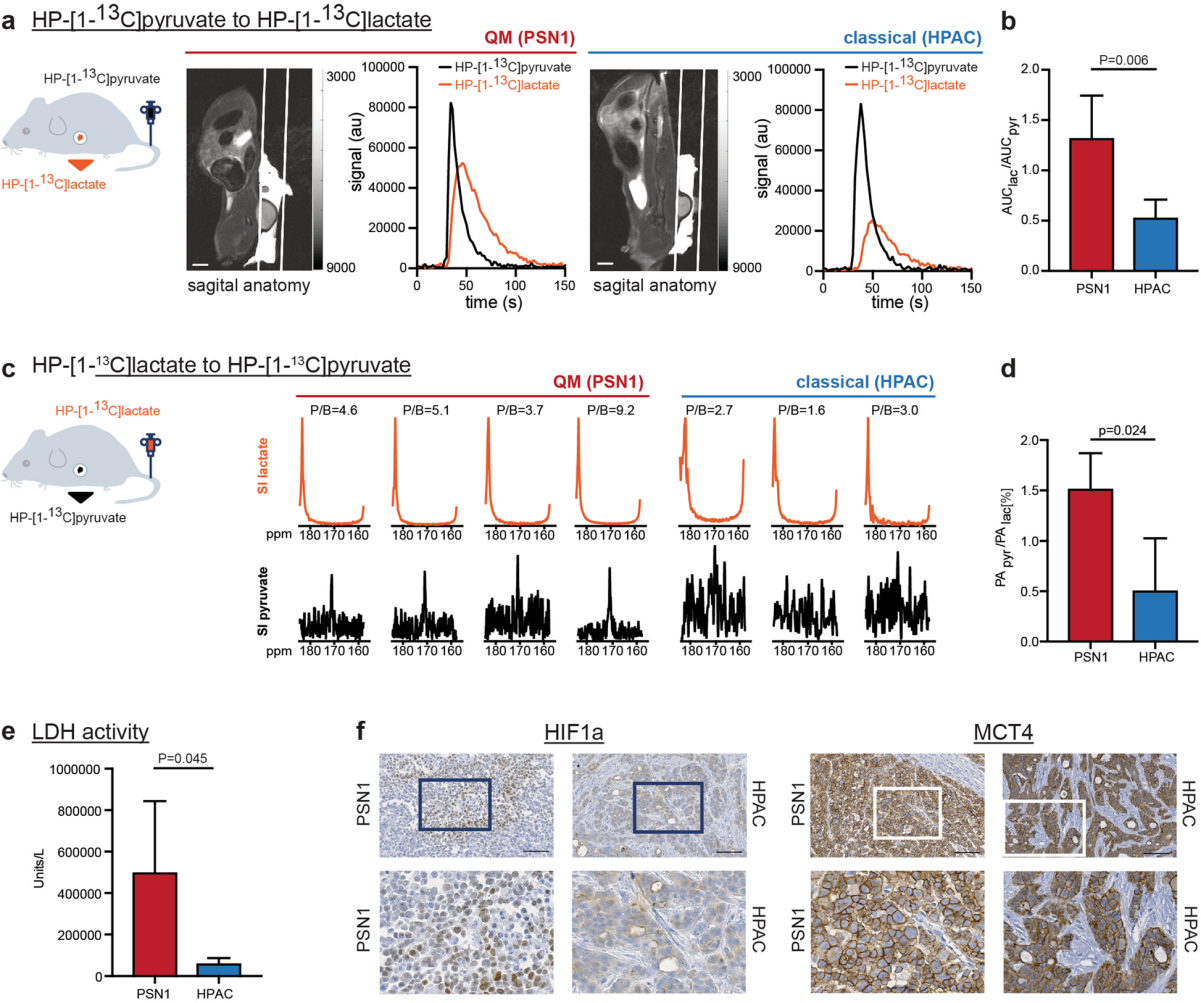
Magnetic resonance spectroscopy (MRS) of HP-[1-^13^C]pyruvate and HP-[1-^13^C]lactate interconversions in PSN1 (QM) and HPAC (classical) PDAC xenografts in rats. **a** Left to right: schematic presentation of HP-[1-^13^C]pyruvate i.v. injection into rats with xenografted PSN1 and HPAC tumors, T2-weighted sagittal anatomical image (scale bar = 1 cm) of a rat bearing a subcutaneous tumor, and graphs demonstrating signal intensity time courses of HP-[1-^13^C]pyruvate and HP-[1-^13^C]lactate measured intratumorally in PSN1 (left) and HPAC (right) rat xenografts. The HP-[1-^13^C]lactate curve (orange) is higher in PSN1 than HPAC xenografts. **b** Calculated relative AUC ratios of HP-[1-^13^C]lactate to perfused HP-[1-^13^C]pyruvate showing higher conversion rate in PSN1 (*n* = 4; 1.325 ± 0.418) than in HPAC tumors (*n* = 5; 0.5349 ± 0.175). **c** Left to right, schematic presentation of HP-[1-^13^C]lactate injected into rats with xenografted PSN1 and HPAC tumors and signal intensity (SI) spectra of perfused HP-[1-^13^C]lactate (top) and detected HP-[1-^13^C]pyruvate (bottom) for PSN1 (*n* = 4) and HPAC (*n* = 3) tumors. The spectra have been summed over 10 time points covering maximum tumor enhancement and normalized to the lactate signal. Higher peak-to-background ratios (P/B 3.7–9.2) were observed in PSN1 tumors in comparison with P/B ratios in HPAC tumors (P/B 1.6–3.0). **d** Signal intensity quantification: PApyr/PAlac ratios are significantly higher in PSN1 (1.49 ± 0.30, *n* = 4) than in HPAC tumors (0.51 ± 0.51, *n* = 3). PA, peak area. All *P*-values in this figure calculated by Student’s *T*-test (unpaired, two sided). **e** Ex vivo measurements of lactate dehydrogenase activity in imaged tumor sample. Higher activity in PSN1 (*n* = 5; 501,794 ± 341,920 U/L) than in HPAC tumors (*n* = 5; 62,796 ± 24,641 U/L) detected. **f** Representative immunohistochemistry for HIF1a and MCT4 in rat PSN1 (*n* = 4) and HPAC (*n* = 2) xenografted tumors. HIF1A-specific nuclear staining was detected exclusively in PSN1 (QM) tumor. MCT4 staining intensity only lightly stronger in PSN1 than in HPAC tumor. Scale bar, 100 µM

Pharmacological inhibition suggested efficacy of GNE-140 in glycolytic cells arguing for the need of unequivocal identification of highly glycolytic PDACs for successful metabolic targeting. However, detection of dominant metabolic pathways driving tumor phenotypes remains a highly challenging task. Fluorodeoxyglucose (^18^F-FDG) positron emission tomography (PET) is clinically established method for tumor detection based on high glucose uptake into the cancer. However, ^18^F-FDG-PET detects only the very first step of glycolysis since ^18^F-FDG gets phosphorylated by the first glycolytic enzyme hexokinase or glucokinase and does not enter the further metabolic processing. Thus, FDG-PET detects the glucose trapping in the cell, rather than the real glycolytic activity of the tumor. We sought to evaluate the last step of glycolysis, pyruvate to lactate conversion, and explored HP-MRS with [1-^13^C]pyruvate and [1-^13^C]lactate in PDAC in an in vivo approach. For this purpose, rats were subcutaneously implanted with glycolytic QM PSN1 or classical HPAC PDAC cell lines. Consistent with the respective molecular subtype, PSN1 tumors presented an undifferentiated mesenchymal histology, while HPAC tumors showed a more differentiated epithelial tumor (supplementary Figure 3a). Once the tumors reached a minimal size of 5 × 5 mm, metabolic spectroscopy was performed. HP-[1-^13^C]pyruvate was i.v. injected into the tail vein, and intra-tumoral accumulation of HP-[1-^13^C]lactate was followed in real time. Using MRS, significantly more HP-[1-^13^C]lactate was detected in PSN1 compared to HPAC tumors, supporting higher ^13^C label exchange between pyruvate and lactate specifically in PSN1 tumors (Fig. 5 a and b, supplementary Figure 3b).

To evaluate whether lactate is imported and used by tumors in vivo as observed in vitro in Seahorse experiments, we also performed the reverse experiment and intravenously injected HP-[1-^13^C]lactate in PSN1 and HPAC tumor rats in vivo. High HP-[1-^13^C]lactate uptake and intratumoral HP-[1-^13^C]pyruvate were clearly detected in PSN1 compared to very low HP-[1-^13^C]pyruvate signal in HPAC xenografts (Fig. 5c, supplementary Figure 3b). Accordingly, significantly higher peak area (PA) PApyr/PAlac ratios were measured for PSN1 than HPAC tumors (Fig. 5d).

Taken together, highly glycolytic PSN1 xenografts could readily be discriminated based on high HP-[1-^13^C]pyruvate to HP-[1-^13^C]lactate interconversion observed in HP-MRS.

Lactate dehydrogenase (LDH) enzymatic activity measured ex vivo after the spectroscopy experiment in snap frozen tissues was also higher in PSN1 compared to HPAC tumors (Fig. 5e) consistent with the in vivo finding. We further confirmed the highly glycolytic nature of rat PSN1 tumors by immunohistochemical analysis of glycolytic markers HIF1A and MCT4 in rat PSN1 and HPAC xenografts used in MRS experiments. MCT4 showed the typical membrane-associated expression in cancer cells in both xenografts, with only slightly stronger staining intensity in PSN1 than in HPAC tumors (Fig. 5f) but not prominently different. Intriguingly, staining for HIF1A, a major glycolytic regulator in cancers, was found exclusively in the PSN1 tumors with typical nuclear expression pattern in the cancer cells (Fig. 5f). Next, we also analyzed HIF1A and MCT4 expression in murine xenografts of human PDAC cell lines (supplementary Figure 4). Indeed, MCT4 staining intensity was also only lightly stronger in the murine QM xenografts compared to xenografts of classical cell lines. Specific nuclear HIF1A expression was limited to QM tumors only (PSN1, KP4, MIAPaCa2, PaTu8988T), and not detected in classical tumors (HPAFII, PaTu8988S, HUPT4, HPAC) (supplementary Fig. 4).

## Discussion

The challenge in PDAC is its enormous therapy resistance due to the evolution of aggressive cancer cells driven by oncogenic KRAS and loss of key tumor suppressors in a complex adapting microenvironment with various signaling effectors and biophysical and hypoxic restraints. Despite considerable genetic homogeneity with regard to oncogenic KRAS as lead driver, many studies support the existence of several molecular PDAC subtypes, including classical/progenitor and QM/squamous/basal-like and hybrid states with more or less pronounced subtype-specific transcriptional programs [3–5]. Though indisputably present, functional aspects and phenotypic cues of the defined transcriptional subtypes are less described. One key feature of PDAC is the metabolic rewiring that may lead to phenotypic features not entirely captured by transcriptomic signatures. PDACs identified as transcriptionally glycolytic show amplification of *KRAS* and *MYC* genes and are associated with a worse prognosis both in resectable and metastatic setting [11]. In patients, targeting of KRAS-MEK-MAPK pathway in a monotherapy approach only is not successful potentially due to activation of escape routes such as PI3K-AKT. However, blocking glycolysis with 2-deoxyglucose in combination with MAPK inhibitor is at least in mice effective and leads to apoptosis induction and reduction in tumor volume, suggesting high potential of this co-targeting [12]. Functional identification of patients with highly glycolytic PDACs can lead clinical decision-making and introduction of anti-glycolytic drugs in the clinic. In this work, we pursued two aims: (i) to evaluate whether the presence of glycolytic transcripts is indeed translated into operable glycolysis in PDAC QM subtype and (ii) offer a noninvasive imaging-based approach for detection of highly glycolytic tumors. We focused this analysis on patient-derived model systems including PDX and PDCs to value the molecular and metabolic heterogeneity in primary PDAC model systems. Furthermore, all our metabolic assays are performed under supplementation with physiological levels of glucose (5 mM), thus omitting the metabolic artifacts that can be caused by the usage of typical high glucose media.

Gene expression analysis in four different model systems (cell lines, PDC, PDX, and bulk tissue samples) indeed identified glucose metabolism/glycolysis/hypoxia/MYC targets as dominating metabolic transcripts of the QM subtype. This is in line with the previously observed “glycolytic” subtype in mesenchymal PDAC cell lines [22] and the recently reported “glycolytic” transcriptional PDAC subtypes in patients [11]. However, glycolysis was not unambiguously functionally dominant in all cells of QM subtype, being cell lines or primary. In functional assays, we observed notable heterogeneity in metabolic behavior especially in patient-derived cells. We found active functional glycolysis in single representatives of the QM subtype, such as PSN1 and PDC69 cells. In Seahorse assays, these cells demonstrated high ECAR to OCR ratios, suggesting that cell intrinsic energy metabolism relies rather on glycolysis than on OXPHOS. It should however be noted that Seahorse assays evaluate ECAR and OCR values in in vitro conditions and are highly dependent on cell culture features such as current cellular density, growth pattern, cell cycle, and current mitochondrial number [26] and should be interpreted only as indication of the cellular energetic status. Glycolytic energetic status of PSN1 and PDC69 correlated well with high gene expression of the lactate producer and transporters LDHA and MCT1/4, respectively, supporting the translation of transcripts in active glucose metabolism. Interestingly, HIF1A, a major transcriptional regulator of glycolysis and cellular response to hypoxia [23], was also well expressed in the identified glycolytic cells here grown in typical in vitro normoxic conditions, supporting intrinsic gene expression programs well preserved in QM cells. Furthermore, by using multiplex immunofluorescence approach, we observed MCT4 expression in KRT81-positive cells in human PDAC samples, further suggesting correlation of QM subtype and glycolytic phenotype. In line with our observations, MCT4 has already been suggested as marker of glycolytic PDACs with poor prognosis [24].

The heterogeneity observed in our results suggests that rigid transcriptome-based classification of PDAC subtypes may not be sufficient as the basis for clinical decisions regarding metabolic targeting approaches. Rather, individual PDACs may often present a continuum of different metabolic states that are more or less phenotypically presented depending on various cell-autonomous and non-cell-autonomous cues. Hybrid PDAC subtypes with transcriptomic signatures in between the classical and QM/basal-like states have been highlighted recently [4, 11]. Similar to our study, a correlation of molecular cues and functional oxidative phosphorylation was very recently reported for PDAC cells [27]. The authors emphasize on metabolic heterogeneity and flexibility and shifts from OXPHOS or glycolysis when necessary, supporting the existence of plastic metabolic states dependent on the environmental challenges. It is reasonable to assume that among PDAC cells, a whole spectrum of weakly to highly glycolytic QM PDAC cells exists. The exclusive dependency on the one or the other metabolic pathway is an unlikely scenario. However, individual tumors with high activity of specific metabolic pathway may exist, and their identification will be the key to successful targeting. We show here that both PSN1 and HPAC xenografted tumors import HP-[1-^13^C]pyruvate, however show different conversion rates to HP-[1-^13^C]lactate. Glycolytic PSN1 tumors were readily detectable by HP-MRS due to higher ^13^C-label exchange among pyruvate and lactate, indicating high activity of the last glycolytic enzyme LDH and high intratumoral pyruvate to lactate conversion. Similarly, in breast cancer patients, high HP-[1-^13^C]pyruvate to HP-[1-^13^C]lactate conversion rates identified strongly glycolytic aggressive triple negative breast cancer with high HIF1a and MCT1 tissue expression [28] and high-grade lesions in prostate cancer with increased MCT4 expression [29]. This approach is already being used in personalized therapy monitoring in prostate and brain cancer [30, 31]. Additionally, alanine-to-lactate signal ratio upon injection of HP-[1-^13^C]pyruvate has also been shown to distinguish well between preneoplastic lesions and PDAC in mouse models [32, 33]. In our study, we focused on potentials of imaging pyruvate-to-lactate interconversions for detection of a subpopulation of highly glycolytic PDACs.

Furthermore, we also confirmed in vivo that HP-[1-^13^C]lactate enters the PSN1 and HPAC tumors, with however lower uptake in classical HPAC xenografts. As a result, we clearly detected HP-[1-^13^C]pyruvate in PSN1 tumors and very low levels in HPAC tumors, what suggests potential subsequent use of pyruvate in the TCA cycle and high metabolic flexibility of PSN1 tumors for pyruvate and lactate as oxidative fuels. Lactate has recently been considered as one of the important actors in tumor metabolism [34]. Tumors use the advantage of lactate being the second most abundant metabolite in the systemic circulation and readily feed the TCA cycle with pyruvate generated from lactate in a reverse lactate dehydrogenase reaction [25, 35, 36]. Indeed, in Seahorse experiments, we also observed OXPHOS activation with lactate in PDAC cells, especially in the QM cell lines. However, it should be noted that Seahorse experiments were performed under deprivation of main OXPHOS fuels, glucose and glutamine. This may potentially lead to overemphasis of lactate usage in OXPHOS under these in vitro conditions.

We hypothesize that the hypoxic microenvironment of the tumor favors the epithelial-to-mesenchymal transformation (EMT) of the cancer cells and appearance of the glycolytic QM tumors. These tumors potentially adapt their oxidative metabolism to fuels which are then locally produced, either by themselves or by neighboring cancer, stromal, or immune cells. In some cases, this will likely be through oxidation of lactate, although lactate oxidation by itself may not be a sufficient marker for the classical to QM transition.

Our HP-MRS experiments provide evidence for the concept that PDACs with high reliance on glycolysis are potentially detectable via HP-[1-^13^C]pyruvate/lactate MRS imaging (MRSI) in clinical practice. Thus, identification of highly glycolytic, aggressive PDACs by HP-[1-^13^C]pyruvate and HP-[1-^13^C]lactate MRSI may be used to guide and monitor tumor treatment with anti-glycolytic therapies. The limitation for clinical translation of this method is the low pyruvate SNR measured after the HP-lactate injection, leading to an uncertainty on the performed quantifications. Therefore, future improved methods of higher sensitivity of the HP-[1-13C]lactate experiment such as employment of cryogenically cooled receiver coils would be of high interest and may even allow to quantify metabolic fluxes within the different subtypes of PDAC.

## Conclusion

In contrast to biopsy-based tumor characterization, metabolic imaging allows dynamic evaluation of the whole tumor limiting sampling bias and addressing tumor heterogeneity [37]. Although likely not all QM tumors are potentially extremely glycolytic, noninvasive detection of highly glycolytic PDACs by HP-[1-^13^C]pyruvate/lactate MRS is one of the first methods for successful individual metabolic approaches.

## Supplementary Information

**Additional file 1.**

**Additional file 2.**

**Additional file 3.**

**Additional file 4.**

**Additional file 5.**

## Data Availability

All data generated in this paper are available from the corresponding author upon a reasonable request.

## Abbreviations

PDAC: Pancreatic ductal adenocarcinoma
QM: Quasi mesenchymal
HP-MRS(I): Hyperpolarized magnetic resonance spectroscopy (imaging)
PDX: Patient-derived xenografts
FFPE: Formalin-fixed paraffin embedded
PDC: Patient derived cells
MCT4: Monocarboxylate transporter 4
MCT1: Monocarboxylate transporter 1
LDH: Lactate dehydrogenase
HIF: Hypoxia-inducible factor
NES: Normalized enrichment score
FDR: False discovery rate
OCR: Oxygen consumption rate
ECAR: Extracellular acidification rate
TCGA: The Cancer Genome Atlas
DMEM: Dulbecco’s Modified Eagle Medium
RPMI: Roswell Park Memorial Institute
OXPHOS: Oxidative phosphorylation
^18^F-FDG: 18F-fluorodeoxyglucose
PET: Positron emission tomography
