# Supplementary material for: Functional noninvasive detection of glycolytic pancreatic ductal adenocarcinoma
Source: Cancer Metab. 2022 Dec 9;10:24. doi: 10.1186/s40170-022-00298-5 (PMC9737747; doi:10.1186/s40170-022-00298-5)
Supplement: Supplementary file 1 — Additional file 1. [file 40170_2022_298_MOESM1_ESM.docx]

**Supplementary material and methods**

**Cell culture**

**PDAC cell lines**

All PDAC cell lines have been obtained from the ATCC and regularly externally authenticated by Multiplexion (at least once a year). PDAC cell lines (Psn1 (RRID: CVCL_1644); Kp4 (RRID: CVCL_1338), PaTu8988T (CVCL_1847); MiaPaca2 (RRID: CVCL_0428), PaTu8988S (RRID: CVCL_1846), HPAC (RRID: CVCL_3517), HPAFII (RRID: CVCL_0313), and HupT4 (RRID: CVCL_1300) were grown in Dulbecco’s Modified Eagle Medium (1:1 mix of DMEM, #11966025 and #A1443001, Thermo Fisher Scientific, Waltham, USA) adapted to final concentrations of 5 mM D-glucose (Thermo Fisher Scientific, Waltham, USA), 2 mM L‑glutamine, 5% v/v fetal bovine serum (FBS, Thermo Fisher Scientific, Waltham, USA), and 1% v/v penicillin/streptomycin (P/S, Thermo Fisher Scientific, Waltham, USA) if not stated otherwise. The cells were incubated at 37°C and 5% CO_2_, provided with fresh medium every 2-3 days, and passaged at a confluency of 80‑90%.

### Patient Derived Cells (PDCs)

From 11 PDX samples, we were able to isolate and cultivate primary patient derived cells (PDCs) for further analysis. After explantation, tumor tissue was stored in RPMI (Thermo Fisher Scientific, Waltham, USA) without any supplements on ice. The tumor tissue was then minced on ice into 1-2 mm³ cubes. The minced tissue was incubated at 37°C in digestion solution (RPMI containing 5 mg/mL Collagenase II (Thermo Fisher Scientific, Waltham, USA) and 1.25 mg/mL dispase (Thermo Fisher Scientific, Waltham, USA) with agitation to dissociate the tumor tissue. Cell suspension was subsequently filtered through a 100 µm mesh and cells were collected by centrifugation (300 x g, 5 min) at room temperature. The cell pellet was washed with media and cultivated in growth media consisting of a 1:1 mixture of Keratinocyte-SF medium and RPMI 1640 (Thermo Fisher Scientific, Waltham, USA). Cells were cultured on collagen coated dishes (Corning B.V. Life Science, Amsterdam, Netherlands) in a humidified incubator at 37 °C with 5% CO2. To obtain pure PDAC cell line, cells were treated with differential trypsinization until no contaminating fibroblasts were detected by visual inspection under the microscope. The human character of the lines was confirmed by STR analysis. Established cells were then further cultivated on standard cell culture dishes.

For all metabolic analysis, PDC cell lines were further cultivated in a 1:1 mixture of Keratinocyte-SF medium (#17005075, Thermo Fisher Scientific, Waltham, USA) and RPMI 1640 (#11879020, Thermo Fisher Scientific, Waltham, USA) adapted to final concentrations of 5mM D-glucose, 4.5mM L-glutamine, 0.26mM sodium pyruvate, and 6%v/vFBS, and 1% v/v mixture of penicillin/streptomycin (P/S, Thermo Fisher Scientific, Waltham, USA) and Antimycotic/Antibiotic (cat. nu. 15240-062, Thermo Fisher Scientific)if not stated otherwise. The cells were incubated at 37°C and 5% CO_2_, provided with fresh medium every 2-3 days, and passaged at a confluency of $\sim$70-90%. The primary cells were usually used 6-25 passages after thawing.

**PDX samples preparation**

Establishment of the PDX mouse model was performed using surgically resected PDAC tissues collected from patients at the Ruhr-University Bochum Comprehensive Cancer Center. Informed and written consent was obtained from all patients. The study was approved by the ethics committee of the Ruhr University Bochum (permission no. 3534-9, 3841-10, 16-5792). Patient tumor tissues were xenografted in both flanks of nude mice and expanded, isolated and re-implanted for at least three generations. All animal experiments were performed according to the guidelines of the local Animal Use and Care Committees at the Ruhr University Bochum (8.87-50.10.32.09.018, 84-02.04.2012.A328  and 81-02.04.2017.A423).

**RNA isolation and gene expression analysis**

Established/PDC cells were cultivated for 48 hours in the respective media. At confluence of 70-80%, cells were placed on ice and washed twice with ice-cold PBS, mechanically scratched from the plate in 1ml of ice-cold PBS and centrifuged at 4°C/400g for 5 minutes. Pelleted cells were stored in -80^⭘^C till all cells were collected for RNA isolation. RNA was isolated using the Maxwell RSC simplyRNA Cells Kit (#AS1390, Promega, Germany). Cell RNA isolation kit according to the manufacturer’s instructions. Total RNA was stored at -80°C till further processing and gene expression analysis. For PDX samples, RNA was isolated from fresh frozen PDX tumor tissue using the PARIS (Ambion) isolation kit.

For PDC and PDX samples, RNA samples were used for gene expression profiling performed using the HT12-v4 expression Bead Chip (Illumina.com) at the Genomic Core Facility at DKFZ Heidelberg. Bead intensity information was extracted using the Bioconductor package illuminio and the limma package was used for background correction. Using the necq function, quantile normalization was performed followed by log transformation. Low expression features were filtered and the probes were collapsed to the highest. The normalized expression values were then exported for downstream analysis.

For PDAC cell lines, RNA-seq analysis was performed at CeGaT GmbH. Gene expression was achieved by means of 100 bp paired end mRNA sequencing on an illumine Novaseq 600. Reads were preprocessed using casava, cutadapt and Skewer v.0.2.2 and mapped to the hg38 reference genome using STAR v.2.7.1a. Quantification was performed during alignment using STAR with the –quantMode GeneCounts parameter. A gene expression count matrix was generated with raw read counts using edgeR and stored in s DGEList object containing sample phenodata. All genes with less than 2 count per million read (cpm) in more than 20% of all samples considered were filtered out. Reads were normalized using the normalization function in edgeR and the normalized read count matrix was used for downstream analysis.

**Bioinfomatic platform for subtype determination**

For subtype determination, the normalized expression values were imported in R and the top 3000 most variable features were then selected by calculating the median absolute deviation. The selected features were centered relative to the median and unsupervised clustering was performed using the concensus clusterplus algorithm and validated using a non-negative matrix factorization approach. Gene set enrichment analysis was performed to determine the subtype of each samples by comparing with data from previously published pioneering subtyping studies (1,2).

To compare QM and classical mRNA data sets, Gene Set Enrichment analysis was performed using the Broad Institute GSEA software (3) , version 4.0.

Human bulk PDAC gene expression data has been previously described (4) (E-MTAB-1791).

Sequencing and gene expression files for cell lines, PDC and PDX samples have been uploaded to the Gene Expression Omnibus (GEO) and have following accession numbers: Cell lines (E-MTAB-10765); PDC (E-MTAB-10763); PDX (E-MTAB-10784).

**Seahorse metabolic flux assays**

Cell lines were seeded in a seahorse 96 well microplate in 80µL final volume in respective media mentioned above and cultivated overnight in cell culture incubators (5%CO_2_, 37°C). Metabolic flux assays were performed the next day. For measurements of ECAR and OCAR levels under supplementation of 5mM glucose and 2mM glutamine, cell culture media was exchanged 1 hour prior to measurement with respective Seahorse media (Seahorse XF DMEM or Seahorse XF RPMI medium, Agilent Technologies, Santa Clara, USA) supplemented with 5mM glucose and 2mM glutamine (Agilent Technologies, Santa Clara, USA). Plates were incubated 45 minutes to 1 hour in non-CO2 incubator, media was exchanged one more time shortly before the assay and then basal measurements of metabolic status (ECAR and OCR) was performed using the Agilent Seahorse XFe96 machine. Measurement 3 or 4 was usually used for calculation of basal ECAR to OCR ratios. Attention was paid that OCR and ECAR values were are in the optimal measurement range of the instrument (ECAR 10-100, OCR 20-200) and that cells are viable (visual inspection). For proper comparison of OCR and ECAR values among different cell lines, one assay (one Seahorse plate) always contained all 8 commercial cell lines or all 7 PDCs. Assays were repeated as independent experiments at least twice and results showed similar tendencies.

In all assays, 5-6 wells/technical replicates were seeded with cells of one cell line and used for calculation of mean value per cell line. Assays were analyzed using the Wave 2.4.0 software. In figure 2a (ECAR/OCR ratios) presented are mean+SD from 2 independent experiment, for each experiment 5-6 wells (technical replicates) per cell line. Energetic maps are plotted using values from one experiment (mean+SD calculated from 5-6 technical replicates). For higher accuracy of energetic maps, in parallel to 96-well plate used in Seahorse experiment, one more 96 well Seahorse microplate was seeded with cells using the same prepared cell suspensions and was incubated over night. The plate was then washed twice with PBS, each well tripsinized with 20µl 0.05% Tripsin/EDTA for 20 min at 37C, 20µl of Tripan Blue was aded to each well and cells were counted using a Biorad TC20 automated cell counter. The cell counts of viable cells (mean value of 2-4 wells per cell line) were then used for normalization of Seahorse ECAR and OCR data, thus assuring that energy maps represent values measured in viable cells only.

For all seahorse assays, seeding cell densities (in general 15.000-20.000 cells/well) are presented in supplementary table 3.

**Basal energy measurements under glucose/glutamine starvation and lactate supplementation assay**

Cells were seeded in a Seahorse 96 well microplate coated with Cell-Tak cell adhesive in their usual respective cultivation media mentioned above (final volume: 80 µL/well). Cells were seeded in technical triplicates/3 wells per cell line. After over-night incubation in cell culture incubators (5%CO_2_, 37 C), the medium was exchanged for non-supplemented DMEM or RPMI –“basal” media (no glucose, no glutamine) or supplemented with 10 mM L-lactate (sodium L-lactate, Sigma-Aldrich, cat. nu. #L7022). Media were lacking glucose, glutamine or pyruvate but were supplemented with 1% v/v FBS. The cells were incubated in the respective starved/basal or lactate supplemented medium at 37°C and 5% CO_2_ for 6 hours. Prior to the seahorse measurement, medium was changed to Seahorse XF DMEM (cat. num. 103575-100) or Seahorse XF RPMI medium (cat. num. 103576-100 or 103336-100, Agilent Technologies, Santa Clara, USA) either without any supplementation -“basal Seahorse” media or supplemented with or 10 mM L-lactate (sodium L-lactate, Sigma-Aldrich, 867561) -“basal Seahorse+lactate” and the cells were incubated in a non-CO_2_ incubator at 37°C for 45-60 min. For all Seahorse media the pH was adjusted to 7.4 according to the manufacturer’s instructions. Addition of sodium L-lactate to the medium did not change the pH of the media. 6 basal measurements of OCR and ECAR were performed, measurement 3 or 4 used for calculations. For estimation of OCR values under starvation (no glucose, no glutamine), OCR values were normalized to 10.000 of seeded cells/well. In supplementary figure 2b, mean values+SD per cell line calculated from 3 technical replicates in one experiment are presented. The assay was repeated at least twice with similar result observed. For lactate supplementation assay, mean OCR value of lactate-supplemented wells (3 technical replicates) was divided with mean OCR value of non-supplemented/basal wells (3 technical replicates) and OCR-lactate/OCR-basal ratio was obtained for each cell line. The assays were repeated at least twice. In figure 4a, mean+SD values of OCRlactate/OCRbasal ratios in all performed assays are presented (2 independent assays for conventional cell lines, 4 assays for PDCs).

For all seahorse assays, seeding cell densities (in general 15.000-20.000 cells/well) are presented in supplementary table 3.

**Lactate/Glucose concentrations in the media**

Changes in the lactate and glucose concentrations in culture medium of PDAC cell lines were observed over a 24-48-72-96 hour time period. The assay was performed in a 6-well dish format with three technical replicates per cell line and time point. Cells were seeded in respective DMEM media mentioned above (final volume: 1.5 mL/well). At defined time points (24-48-72-96 hours) , medium was collected (600µL) and the cells in the respective wells were tripsinized and collected and cell number determined using the TC20 automated cell counter (BIO-RAD Laboratories, Hercules, USA). Lactate and glucose concentrations in the media were determined in the central laboratory facilities at the university clinic in Essen. Measured lactate concentrations were corrected for lactate concentration present in the media without cells (0.5mmol/l) and for evaporation factor (media evaporation observed after 48-72-96 hours of cultivation). In addition, lactate concentrations were normalized to cell numbers and normalized values are presented in the figure as well.

**Quantitative Real-Time PCR**

RNA isolation was done with Maxwell® RSC simplyRNA Cells Kit (Promega, AS1390) by using Maxwell® RSC Instrument (Promega, AS4500) according to manufactrer’s instructions. Invitrogen™ SuperScript™ IV First-Strand Synthesis System (ThermoFischer Scientific, 18091050) was used to synthesize cDNA. Approximatelly 1-2µg of total RNA was used for every cDNA synthesis. Semi-quantitative PCR was performed with SYBR GREEN PCR Master Mix (Roche, 04707516001). Relative expression of genes of interests from each sample were determined with LightCycler 4800 instrument (Roche, #05015278001). Beta-glucuronidase (*GUSB*) gene were used as reference. Results were calculated using the standard curve and the comparative delta Ct method. Mann-Whitney test was used for statistical analysis. Primers sequences were as follows: GUSBfwd:TGCAGGTGATGGAAGAAGTG and GUSBrvs: TTGCTCACAAAGGTCACAGG; HIF1Afwd:GCCGCTGGAGACACAATCAT and HIF1Arvs: TGGGTGAGGGGAGCATTACA; SLC16A1fwd: CACCAGCGAAGTGTCATGGA and SLC16A1rvs: ATCAAGCCACAGCCTGACAA; SLC16A3fwd: ATCACTGGCTTCTCCTACGC and SLC16A3rvs: CTGTAGCCGATCCCAAACTC;

**Cell viability assays**

Cell viability was determined using the CellTiter-Glo® (CTG) Luminescent Cell Viability Assay (Promega) according to manufacturer’s instruction. Cell numbers (number of cells seeded per well) were optimized for 96-well cell plate format using CTG assay, attention was paid that optimal cell numbers are in linear range of luminescence measurement. Metabolic inhibitor GNE-140 (MedChemExpres) was dissolved in dimethyl sulfoxide (DMSO) (Sigma-Aldrich) and printed in the indicated logarithmic concentration ranges using the D300e Digital Dispenser (Tecan). The DMSO concentration in each well was adjusted to the highest value on the plate which was set to < 0.1% of the assay volume. Sealed plates were frozen at ‑80°C until use. Cells were grown in their respective media and detached by 0.05% trypsin - ethylenediamine tetraacetic acid (EDTA) (1x) (Thermo Fisher Scientific) and recovered by centrifugation. Optimized cell numbers were seeded in 100µl of respective media with the Multidrop Combi Dispenser (Thermo Fisher Scientific) onto the pre-printed plates and incubated at 37°C and 5% CO_2_ for 72 hours. Cell viability was determined using the CellTiter-Glo® Luminescent Cell Viability Assay (Promega) according to manufacturer’s instruction. Shortly, 100µl of CTG reagent (diluted 1:4 in PBS) was added to each well using the Multidrop Combi Dispenser (Thermo Fisher Scientific), plate was shaken for 2 minutes and allowed to settle at room temperature for 10 min. The luminescence signal was measured with a Tecan Spark® 10 M multiplate reader (Tecan). Data were normalized to the signal of DMSO treated cells. IC50 determination was performed using the Graph Pad Prism v. 7 ‘log (inhibitor) vs. normalized response (variable slope)’ equation.

**Immunohistochemistry (IHC) and immunofluorescence**

For immunohistochemistry and multiplex immunofluorescence on human PDAC samples, a cohort of 31 patient samples from Radboud University Medical Center in Nijmegen, the Netherlands was used. Given the retrospective nature of this study and the anonymized handling of data, informed consent was waived by the institutional review board (protocol CMO2018-4420). Immunohistochemistry was performed according to standard laboratory procedures on PFA fixed, FFPE tissue samples. Antibodies used in this study: MCT4, Atlas Antibodies (Sigma Aldrich, Cat#HPA021451, RRID:AB_1853663); HIF1a, BD Transduction laboratories #610959 (RRID:AB_398272); MCT1, Abcam, #ab85021(RRID: AB_10674945); KRT81, Santa Cruz, #sc-100929 (RRID: AB_2132772); panCytokeratin , Abcam #ab6401(RRID: AB_305450). Signals were developed using horseradish peroxidase-DAB detection system (brown signals). Multiplexed IF was performed using the Opal multiplex system (NEL811001KT, Perkin Elmer, MA) according to manufacturer’s instruction. In brief, FFPE sections were deparaffinized and then fixed with 4% paraformaldehyde prior to antigen retrieval by heat-induced epitope retrieval using citrate buffer (pH 6) or Tris/EDTA (pH 9). Each section was put through several sequential rounds of staining; each includes endogenous peroxidase blocking and non-specific protein blocking, followed by primary antibody and corresponding secondary horseradish peroxidase-conjugated polymer (Zytomed Systems, Germany or Perkin Elmer). Each horseradish peroxidase-conjugated polymer mediated the covalent binding of different fluorophore using tyramide signal amplification. Such covalent reaction was followed by additional antigen retrieval in heated citrate buffer (pH6) or Tris/EDTA (pH9) for 10 min to remove antibodies before the next round of staining. After all sequential staining reactions, sections were counterstained with DAPI (Vector lab). Slides were scanned and digitalized by Zeiss Axio Scanner Z.1 (Carl Zeiss AG, Germany) with 10x objective magnification. The whole-slide images were analyzed using digital image analysis software (HALO^TM^ Version 2.0, Indica Labs, Corrales, NM). Total cell number, and the number of MCT4^+^, Krt81^+^, PanCK^+^ cells were quantified in the total fraction of tissue surface area. The following subsets were defined and quantified: Krt81^+^PanCK^+^, Krt81-PanCK^+^, MCT4^+^Krt81^+^PanCK^+^, MCT4^-^Krt81^+^PanCK^+^, MCT4^+^Krt81-PanCK^+^, MCT4-Krt81-PanCK^+^.  Acellular and necrotic areas were excluded from analysis.

**Patient survival analysis**

Gene expression data for MCT4 (SLC16A3) and MCT1(SLC16A1) were extracted from publicly available resource [www.proteinatlas.org](http://www.proteinatlas.org) where RNA-seq data is reported as mean FPKM (TCGA) (5). The best expression cutoff was accepted from the [www.proteinatlas.org](http://www.proteinatlas.org) as well as the Log-rank P-values presented in the figures.

**Hyperpolarized Magnetic Resonance Spectroscopy (HP-MRS)**

**Animal handling and tumor model**

Approval of the animal protection and welfare review board was received prior to study initiation (ROB-55.2-2532.Vet_02-18-91). All experiments were carried out in adherence to pertinent laws and regulations. For all interventions rats ware anesthetized with inhalation of isoflurane 2.5% (v/v) in an oxygen flow rate of 2 l/min, breathing rates and temperature were constantly monitored and kept in standard range (50-70 breathing rate, 37-39 °C) and a tail-vein-catheter was inserted. PSN1/HPAC cells were cultured under standard condition in high glucose DMEM supplemented with 1% MEM Non-Essential Amino Acids Solution , 10% v/v fetal bovine serum and 1% v/v penicillin/streptomycin (all Thermo Fisher Scientific, Waltham, USA). and 1*10^7^ cells were implanted subcutaneously (s.c.) into the back of male or female 6 weeks-old Crl:NIH-*Foxn1^rnu^* rats (Charles River). The experimentator was not blinded for the tumor type in any of the experiments. Tumors of minimum size of 5x5x5 mm^3^ were used for HP-MRS experiments. After euthanization, tumors were removed rapidly and divided into two parts: one was snap frozen in liquid nitrogen, and the other part used for histological tumor sample evaluation.

**Substrates and polarization procedure**

The hyperpolarization of pyruvate was performed as previously described (6). Briefly: 14 M [1-^13^C]pyruvate (Merck, Darmstadt, Germany) supplemented with 15 mM OX063 trityl radical (Oxford Instruments, Abingdon, UK) and 1 mM Dotarem^®^ were polarized with a HyperSense (Oxford Instruments, Abingdon, United Kingdom) for ~60 min at 1.3 K using a microwave frequency of 94.19 GHz and 100 mW power. The sample was dissolved in 3.4 ± 0.3 mL of buffered solution pressurized to 10 bar and heated to 180 °C containing 80 mM TRIS, 0.1 g/l EDTA and 80 mM sodium hydroxide (NaOH) resulting in a 80 mM [1-^13^C]pyruvate solution with mean pH of 6.9 ± 0.4. Apparent *T*_1_ values of 49.2 ± 1.5 s for hyperpolarized [1-^13^C]pyruvate in this injection solution were measured in a 43 MHz Spinsolve carbon benchtop spectrometer (Magritek, Aachen, Germany; Wellington, New Zealand) using a single pulse acquisition with flip angle 10° and TR 5 s.

The hyperpolarization of [1-^13^C]lactate was performed as described before (7). Briefly: 2.21 M of 45-55% w/w [1-^13^C]lactate (Merck, Germany) supplemented with 30% DMSO and 15 mM OX063 trityl radical were polarized using above described instruments and conditioned with a microwave frequency of 94.18 GHz and 100 mW power for ~200 min. Dissolution was performed with 3.1 ml solution of 80 mM Tris/D_2_O/1M NaOD resulting in a 100 mM [1-^13^C]lactate solution with mean pH of 7.3 ± 0.2. Apparent *T*_1_ values of 49.2 ± 2 s for hyperpolarized [1-^13^C]lactate in this injection solution were measured in a 43 MHz Spinsolve carbon benchtop spectrometer (Magritek, Aachen, Germany; Wellington, New Zealand) using a single pulse acquisition with flip angle 10° and TR 5 s.

**MRI Imaging**

All MRI experiments were performed with a small animal 7 T preclinical scanner (Agilent/GE magnet, Bruker AVANCE III HD electronics) with a dual-tuned ^1^H^1^/^13^C volume resonator with inner diameter 72 mm for anatomical proton imaging and ^13^C excitation, and 20 mm diameter ^13^C flexible surface receive coils (RAPID Biomedical). Surface receive coils were placed of top of the s.c. tumors that were covered in Carbopol® 980 (Caesar & Loretz GmbH, Germany) to reduce B_0_ field shimming artefacts. Initial proton localizer images were used to adjust the animal position so that tumors were placed at the center of the magnet and volume resonator. Anatomical proton MR images were acquired using a multi-slice T2-weighted RARE (rapid acquisition with relaxation enhancement) sequence. Axial, coronal, and sagittal images were acquired in order to guide placement of the ^13^C spectroscopy slice on the tumor, without involvement of adjacent tissue. The imaging parameters of the sagittal T2-weighted scans were echo time TE = 30 ms, repetition time TR = 3 s, field of view 128 x 72 mm^2^, in-plane resolution 0.25 x 0.25 mm^2^, 25 slices of 2 mm thickness, and 3 image averages.

Pyruvate-lactate metabolism was measured with multi-frame slice spectroscopy (MRS, 15 mm slice thickness) using alternating metabolite-frequency-selective excitation (flip angle 30°, 250 Hz transmit bandwidth, both metabolites separately excited and measured every 2 s) while injecting hyperpolarized HP-[1-^13^C]pyruvate or HP-[1-^13^C]lactate. Procedure optimization is described in detail in our previous study, which used two of the same animals bearing PSN1 tumors that were used in this study as well (8).

For data analysis after HP-[1-^13^C]Pyruvate injection, lactate and pyruvate spectral peak heights were summed over all time points after signal appeared. The ratio of these areas under curves (AUC_lac_/AUC_pyr_) gives a marker sensitive to conversion of pyruvate to lactate (9).

For data analysis after HP-[1-^13^C]Lactate injection, due to low pyruvate signal, the signal intensities of lactate and pyruvate spectra were averaged over 10 time points near the maximum intensity and then fit with a constant offset plus a Lorentzian function with fixed 30 Hz full-width at half-maximum to determine the peak area (PA), using the least-squares curve fit function in MatLab. A ratio of PA_pyr_/PA_lac_ was used to describe metabolic differences between the groups. Peak to background (P/B) ratios were calculated as peak height divided by mean spectral signal value of 200 spectral points approximately between 157.84 and 168.16 ppm (away from the peak). All statistical analyses for the imaging were performed using Prism 7 (GraphPad Software).

**LDH activity assay**

Ex vivo enzyme measurements of total LDH were performed photometrically on supernatant of 100 µg frozen tumor tissue, shredded in 1 ml of RIPA buffer (1M Tris-HCl (ph 8), 1M NaCl, Nonidet P40, sodium deoxycholate, distilled water) and centrifuged (4°C; 5000 rpm; 15 min) using cobas® c 701/702 system (Roche/Hitachi) following manufacturer’s instructions in the clinical routine laboratory in the department of clinical chemistry of Technical University of Munich.

**Statistical analysis**

All used tests and p-values are indicated on figures or figure legends.

**Data availability**

All data and detailed procedure descriptions are available from the corresponding authors upon a reasonable request.

**References:**

1. Collisson EA, Sadanandam A, Olson P, Gibb WJ, Truitt M, Gu S*, et al.* Subtypes of pancreatic ductal adenocarcinoma and their differing responses to therapy. Nat Med **2011**;17:500-3

2. Bailey P, Chang DK, Nones K, Johns AL, Patch AM, Gingras MC*, et al.* Genomic analyses identify molecular subtypes of pancreatic cancer. Nature **2016**;531:47-52

3. Subramanian A, Tamayo P, Mootha VK, Mukherjee S, Ebert BL, Gillette MA*, et al.* Gene set enrichment analysis: a knowledge-based approach for interpreting genome-wide expression profiles. Proc Natl Acad Sci U S A **2005**;102:15545-50

4. Jandaghi P, Najafabadi HS, Bauer AS, Papadakis AI, Fassan M, Hall A*, et al.* Expression of DRD2 Is Increased in Human Pancreatic Ductal Adenocarcinoma and Inhibitors Slow Tumor Growth in Mice. Gastroenterology **2016**;151:1218-31

5. Uhlen M, Zhang C, Lee S, Sjostedt E, Fagerberg L, Bidkhori G*, et al.* A pathology atlas of the human cancer transcriptome. Science **2017**;357

6. Hundshammer C, Braeuer M, Muller CA, Hansen AE, Schillmaier M, Duwel S*, et al.* Simultaneous characterization of tumor cellularity and the Warburg effect with PET, MRI and hyperpolarized (13)C-MRSI. Theranostics **2018**;8:4765-80

7. Park JM, Josan S, Mayer D, Hurd RE, Chung Y, Bendahan D*, et al.* Hyperpolarized 13C NMR observation of lactate kinetics in skeletal muscle. J Exp Biol **2015**;218:3308-18

8. Topping GJ, Heid I, Trajkovic-Arsic M, Kritzner L, Grashei M, Hundshammer C*, et al.* Hyperpolarized (13)C Spectroscopy with Simple Slice-and-Frequency-Selective Excitation. Biomedicines **2021**;9

9. Hill DK, Orton MR, Mariotti E, Boult JK, Panek R, Jafar M*, et al.* Model free approach to kinetic analysis of real-time hyperpolarized 13C magnetic resonance spectroscopy data. PLoS One **2013**;8:e71996
