# Supplementary material for: Functional noninvasive detection of glycolytic pancreatic ductal adenocarcinoma
Source: Cancer Metab. 2022 Dec 9;10:24. doi: 10.1186/s40170-022-00298-5 (PMC9737747; doi:10.1186/s40170-022-00298-5)
Supplement: Supplementary file 2 — Additional file 2. [file 40170_2022_298_MOESM2_ESM.docx]

***Supplementary figures and tables***

**Supplementary figure 1:**

**
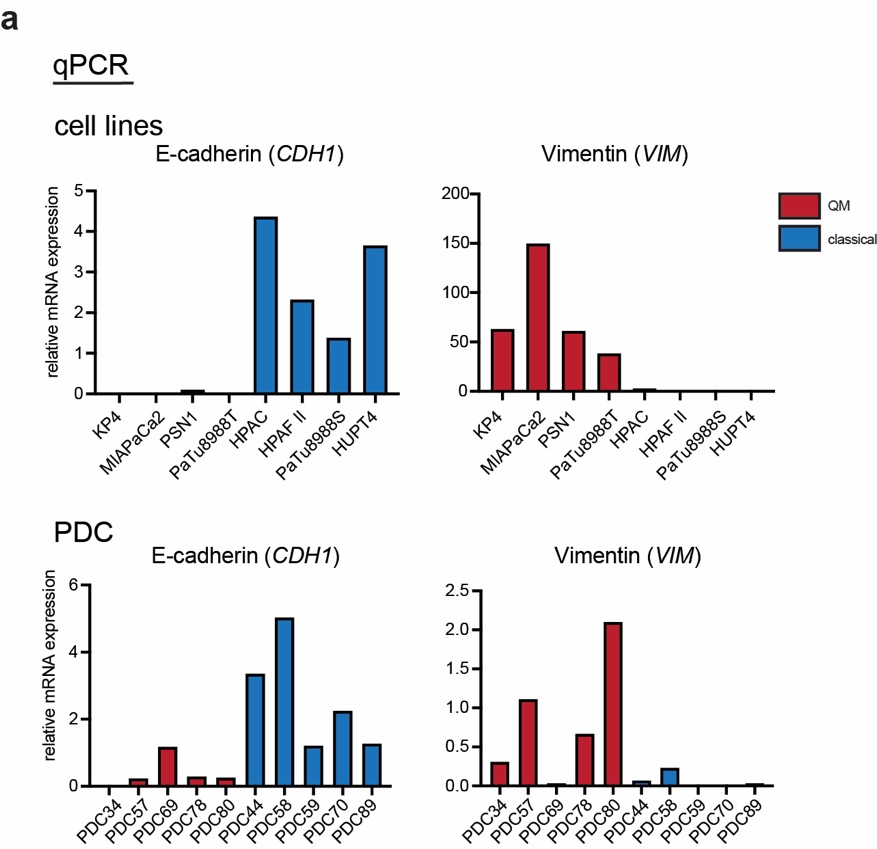
**

**Supplementary figure 1: Gene expression analysis in different PDAC model**

**a)** qPCR analysis of epithelial marker E-cadherin (*CDH1*) and mesenchymal marker Vimentin (*VIM*) in classical and QM PDAC cells. In general, higher expression of E-cadherin in classical and Vimentin in QM cells observed.

**Supplementary figure 2:**

**
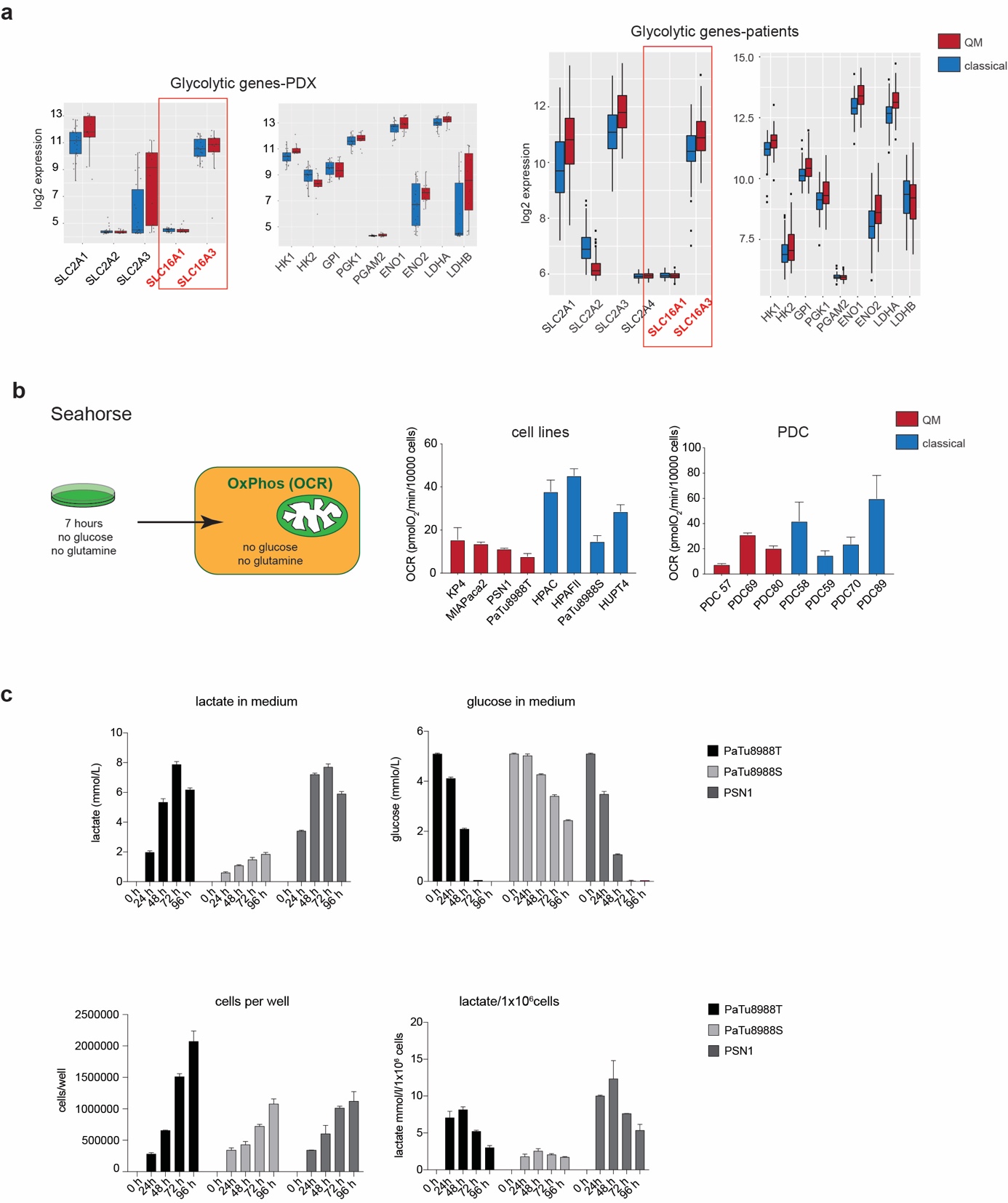
**

**Supplementary figure 2: Glycolysis and lactate metabolism in PDAC. a**) Gene expression analysis for glycolytic genes in PDX and patient PDAC samples (Illumina HT12 bead-array). SLC16A3 presents higher expression than SLC16A1 in both sample cohorts, PDX and bulk patient PDAC. **b)** OCR levels measured for cell lines and PDCs after 7 hours of cultivation in media without glucose or glutamine, “basal media”. For 6 hours cells were in DMEM or RPMI without glucose/glutamine but with 1% FBS, the last 7^th^ hour in Seahorse DMEM or RPMI media without glucose, glutamine or FBS. HPAC, HPAFII, HUPT4 and PDC89 present high relative OCR levels suggesting oxidation of alternative fuels. Presented are OCR values (mean+SD) calculated from 3 technical replicates per cell line in one experiment. OCR values normalized to 10000 seeded cells. At least 2 independent experiments performed; **c)** Measurement of lactate concentrations in the media. PSN1(QM), PaTu8988T (QM) and PaTu8988S (classical) PDAC cells were cultivated in DMEM medium (5mM glucose, 2 mM glutamine, 5% FBS) for 4 days. At defined time points an aliquot of media was collected, cells harvested and counted, and lactate and glucose concentration was measured. In PSN1 and PaTu8988T cells, lactate concentrations first increase (48-72h) corresponding to initial secretion, and then decrease (72h) indicating consumption of locally produced lactate once glucose is deprived. Effects not visible in classical PaTu8988S cells due to slower consumption of glucose. Absolute measured (mmol/l) and cell number normalized (mmol/l/1 million cells) lactate concentrations are presented.

**Supplementary figure 3:**


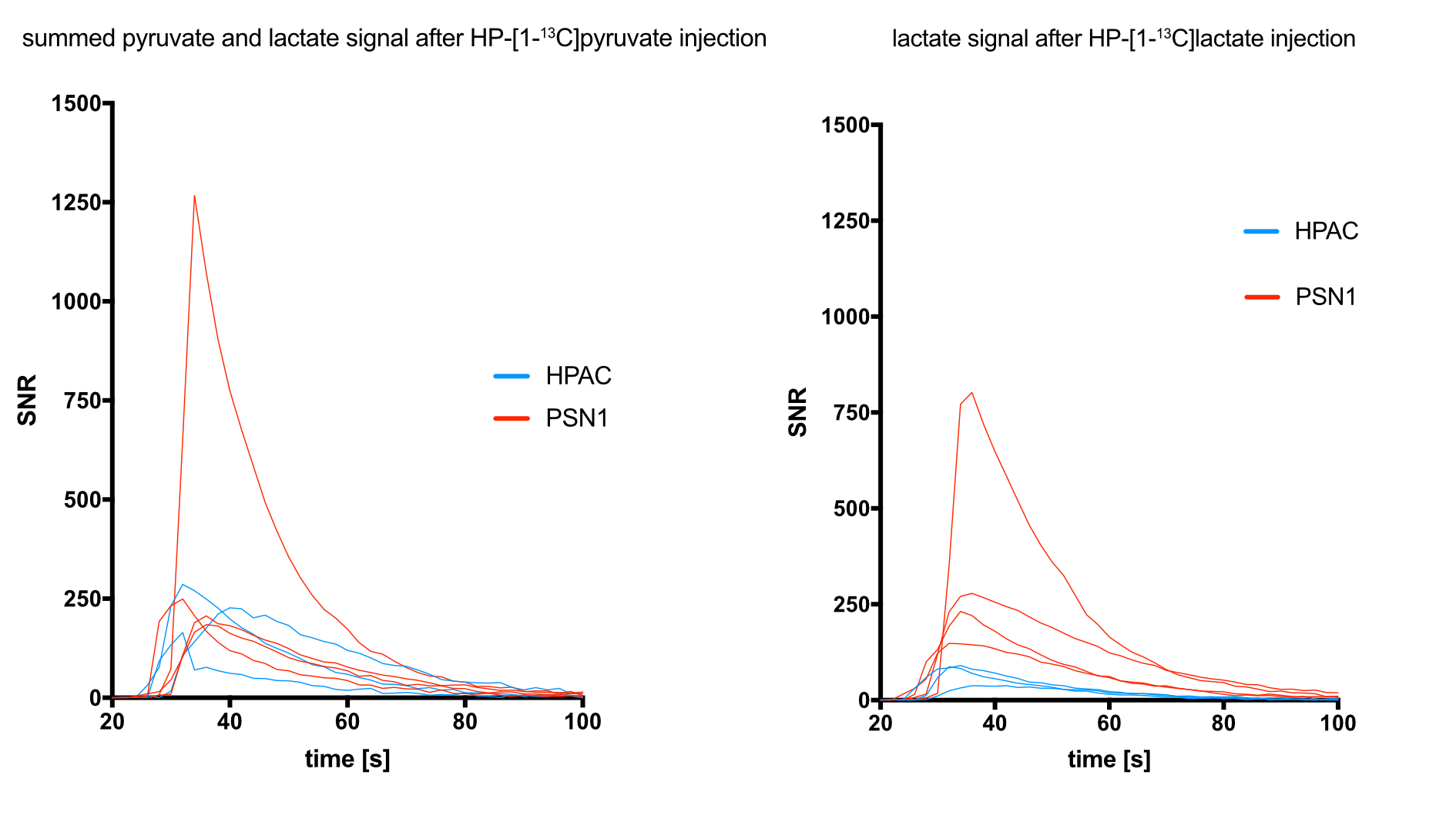
**
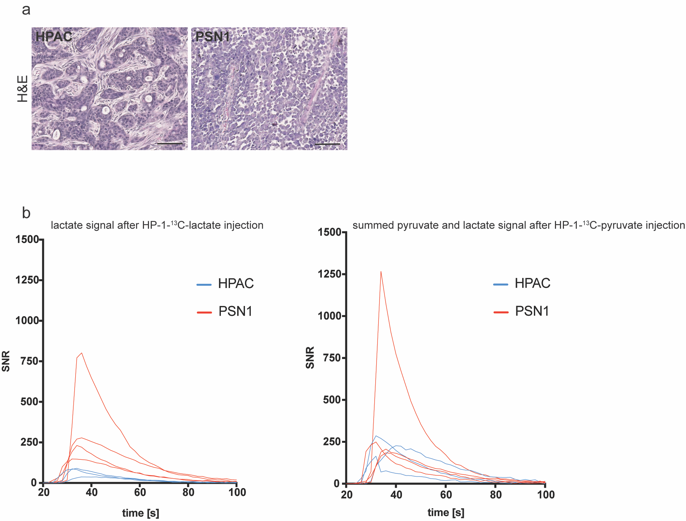
**

**Supplementary figure 3: HP-[1-^13^C]pyruvate/lactate uptake in rat xenografts**

**a**) H&E histological staining of PSN1 and HPAC xenografted tumors used for ^13^C-hyperpolarized HP-MRS in rats. Undifferentiated PSN1 tumors and more glandular, differentiated HPAC tumors. **b)** Left: **_­­­­_**Time curves of SNR of injected HP-[1-^13^C]pyruvate summed up with SNR of converted HP-[1-^13^C]lactate representing the total amount of injected HP-metabolites in animals shown in figure 5A. The sum was used in order to adjust for the different HP-[1-^13^C]pyruvate signal loss due to more rapid conversion rate in PSN1 than in HPAC tumors. Right: **_­­­­_**Time curves of signal to noise ratios (SNR) of HP-[1-^13^C]lactate injected into animals presented in figure 5c. Both injections were preformed within the same imaging session one after the other (first HP-[1-^13^C]lactate, than HP-[1-^13^C]pyruvate) without changes of the animal position or acquisition planning. All surveillance parameters like temperature and beating rate were controlled and kept within the same range between the acquisitions.

**Supplementary figure 4:**

**
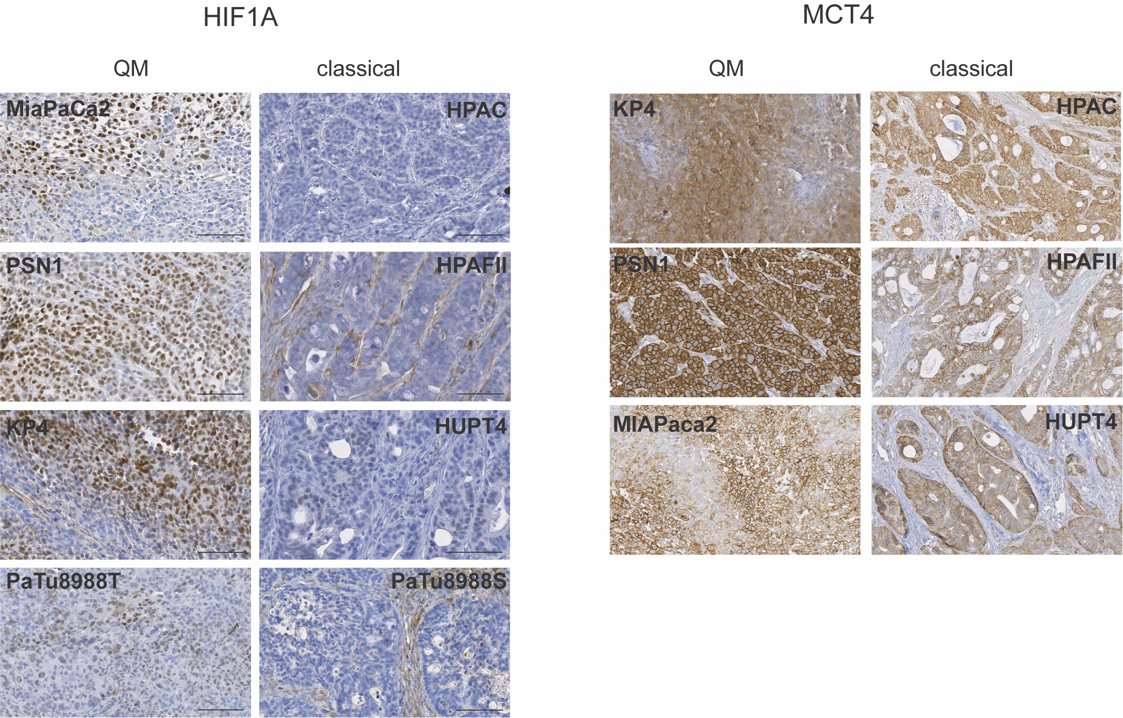
**

**Supplementary figure 4: Immunohistochemistry for HIF1A and MCT4**

Immunohistochemistry for HIF1A and MCT4 on murine xenografted tumor tissues of human PDAC cells lines, one tumor per cell line stained. HIF1A nuclear staining (brown horse radish peroxidase-diaminobenzidine (HRP-DAB) signals) in tumor cells is detectable only in QM xenografts (PSN1, MIAPaca2, PaTu8988T and Kp4). Some HRP-DAB positive signals are detected in the stroma of PaTu8988S and HPAF II xenografts, probably due to non-specific binding of mouse generated HIF1A antibody to murine stroma. Specific membrane-associated MCT4 positive signals are detectable in all tumors, with somewhat stronger signals intensities in PSN1, MIAPaca2 and KP4 QM tumors. Scale bar-100µM.

**Supplementary table 1:**

List of genes used as assigners for the Collisson QM and Bailey squamous subtype (Gene Program 2) used for GSEA analysis **.**

**Supplementary table 2:**

Gene Set Enrichment Analysis-list of all enriched gene sets in established PDAC cells, primary cell, PDX samples and patient cohort. Normalized Enrichment Score (NES) >1.3 and False Discovery Rate (FDR) <0.07 were used as guidelines.

**Supplementary table 3:**

Seeding densities of cells in seahorse experiments.
